# DNA Repair Pathways as Determinants of PARP Inhibitor Resistance and Therapeutic Targets in Cancer

**DOI:** 10.3390/ijms27219814

**Published:** 2026-11-01

**Authors:** Kousalya Lavudi, Malavika Sunita Renjith, Charan Pithana Sai Venkata Vijay, Rekha Rani Kokkanti, Prashant Pandey, Srikanth Ponneganti

**Affiliations:** 1 Department of Radiation Oncology, College of Medicine, The Ohio State University, Columbus, OH 43210, USA; 2 Comprehensive Cancer Center, The Ohio State University, Columbus, OH 43210, USA; 3 Department of Biotechnology, National Institute of Technology Warangal, Hanamkonda 506004, Telangana, India; malavikasr2006@gmail.com; 4 Bioclues.org, Kukatpally, Hyderabad 500072, Telangana, India; venkatacharanvsp@gmail.com; 5 School of Biotechnology, Kalinga Institute of Industrial Technology (KIIT), Deemed to be University, Bhubaneswar 751024, Odisha, India; rekhabioresearch@gmail.com; 6 Department of Oncology, Faculty of Medicine and Dentistry, University of Alberta, Edmonton, AB T6G 2R3, Canada; mrpandeyprashant@gmail.com; 7 Department of Food Science and Technology, The Ohio State University, Columbus, OH 43210, USA; ponneganti.1@osu.edu

**Keywords:** PARPi, homologous recombination, non-homologous end joining, microhomology-mediated end joining, translesion synthesis

## Abstract

Poly (ADP-ribose) polymerase inhibitors (PARPi) have transformed the treatment landscape for homologous recombination-deficient (HRD) cancers, particularly those harboring BRCA1/2 mutations, including breast, ovarian, prostate, and pancreatic cancers, by exploiting the synthetic lethality induced by HR deficiency (HRD) and utilizing HRD status as a predictive biomarker. Nevertheless, intrinsic as well as acquired resistance limits their long-term effectiveness. Resistance often develops as a result of several complex mechanisms including the restoration of homologous recombination (HR), stabilization of the replication fork, enhanced drug efflux, loss of PARP1 expression, epigenetic modifications, and changes in the DNA damage response signaling. BRCA1/2-mutant cells, deficient in HR, become reliant on alternative repair pathways like classical non-homologous end joining (c-NHEJ) and microhomology-mediated end joining (MMEJ). Emerging evidence indicates the importance of replication-induced gaps in single-stranded DNA (ssDNA) and alternative pathways of DNA repair in defining PARPi sensitivity and resistance. This review aims to provide a comprehensive analysis of the established and emerging molecular mechanisms of PARPi resistance, and compensatory DNA repair pathways such as MMEJ. In addition to highlighting resistance pathways, the review also discusses strategies focusing on targeting repair mechanisms and replication stress signaling by using novel drugs targeting DNA polymerase theta (POLQ). It subsequently discusses preclinical advancements and ongoing clinical trials. Through linking the molecular determinants of resistance to the translational status and limitations of the latest therapies, this review offers a comparative framework for understanding resistance mechanisms and strategies to restore PARPi sensitivity in cancers with HRD.

## 1. Introduction

The maintenance of genomic integrity is imperative for normal cellular function and the accurate transmission of genetic information across cell divisions. The eukaryotic genome is continuously threatened by a variety of endogenous and exogenous sources of DNA damage [1]. Endogenously, the production of reactive oxygen species (ROS) and reactive nitrogen species (RNS) can oxidize DNA bases, generate abasic sites and promote single-strand breaks (SSBs) [2]. Exogenously, agents such as ultraviolet (UV) and ionizing radiation (IR) can induce DNA lesions, including pyrimidine dimers, which distort the DNA helix, blocking replication and transcription [3,4]. Over time, the accumulation of DNA damage is a key contributor to cellular aging [5]. To preserve genomic integrity and ensure proper cellular function, cells have evolved a highly coordinated network of DNA damage response (DDR) pathways that detect lesions and coordinate their repair [6].

DNA replication is the process by which the cell duplicates its genomic DNA in order to prepare for cell division during the S phase of the cell cycle. The replication mechanism is, however, not foolproof, and DNA damage can occur if the errors that occur during replication are left unchecked. Upon detection of DNA damage, cells activate checkpoint kinases, particularly ATM and ATR, that phosphorylate downstream effector proteins to arrest the cell cycle, providing time for repair before cell division proceeds [4]. Different types of DNA lesions are rectified by specialized repair pathways. SSBs arise as intermediates during base excision repair (BER) or directly from oxidative attack on the DNA backbone [7]. SSBs are sensed by PARP1 and repaired through the single-strand break repair (SSBR) pathway, involving XRCC1, DNA polymerase β, and DNA ligase 3 [7]. Unresolved SSBs can be converted into DSBs upon colliding with the replication fork [8]. DSBs are the most cytotoxic of all DNA lesions and can be repaired by non-homologous end joining (NHEJ), which is active throughout the cell cycle but is error-prone, or by homologous recombination (HR), which uses a homologous DNA sequence, typically the sister chromatid, as template and is thus restricted to the S and G2 phases of the cell cycle [9].

The fidelity of HR depends on a set of tumor suppressor proteins, mainly BRCA1 (Breast Cancer gene 1) and BRCA2 (Breast Cancer gene 2), which are essential for the nucleation and stabilization of the RAD51 recombinase at resected DSB ends [10]. When BRCA1/2 are mutated, the HR pathway is significantly impaired, and the cells have to rely on alternative repair mechanisms, such as NHEJ, microhomology-mediated end joining (MMEJ), and single-strand annealing (SSA), that tend to be more error-prone, creating a state of continuous genomic instability [11]. This HR deficiency can predispose individuals to breast, ovarian, pancreatic, and prostate cancers [12,13]. Apart from BRCA1/2, dysregulation of other HR factors such as RAD51 and PALB2, as well as activation of proto-oncogenes and inactivation of tumor suppressor genes, can impair the cell’s ability to maintain genomic fidelity [14].

The development of PARP inhibitors (PARPi) is the best example of the therapeutic exploitation of HR deficiency. PARP1 identifies SSBs and facilitates repair by recruiting downstream repair factors following auto-PARylation. PARPi block PARP1 catalytic activity and also lead to PARP1 trapping on damaged DNA, resulting in cytotoxic PARP-DNA complexes that stall replication forks and ultimately create DSBs [15]. Since these DSBs cannot be repaired by HR in BRCA1/2-deficient cells, this failure leads to cell death. This phenomenon is termed synthetic lethality [16]. Several PARPi, such as olaparib, rucaparib, niraparib and talazoparib, have been approved for the treatment of BRCA-mutated cancers. However, the clinical benefit of PARPi is limited by the emergence of resistance through mechanisms such as restoration of HR capacity via secondary BRCA1/2 reversion mutations, loss or alteration of PARP1 itself, upregulation of drug efflux transporters and engagement of alternative DNA repair pathways such as MMEJ [17,18]. Understanding the DNA repair pathways that govern PARPi sensitivity and resistance is therefore a central challenge in cancer therapeutics. PARPi response and the mechanisms of resistance vary across tumor types and molecular backgrounds, as discussed in a later section [19,20].

This review provides a comprehensive and focused examination of DNA repair pathways that determine PARPi sensitivity and resistance, with emphasis on the mechanistic basis of resistance and the therapeutic strategies to overcome it. By including recent advances in our understanding of HR, NHEJ, translesion synthesis (TLS) and MMEJ, we aim to provide insights into emerging treatment modalities that focus on various DNA repair pathways, specifically POLQ, by considering preclinical data, current clinical trials, and limitations of the approaches used. Overall, the article tries to provide a comprehensive perspective on the possibility of these methods in overcoming resistance to PARPi.

## 2. Targeting DNA Repair: A Longstanding Interest in Cancer Therapy

**Figure 1:**
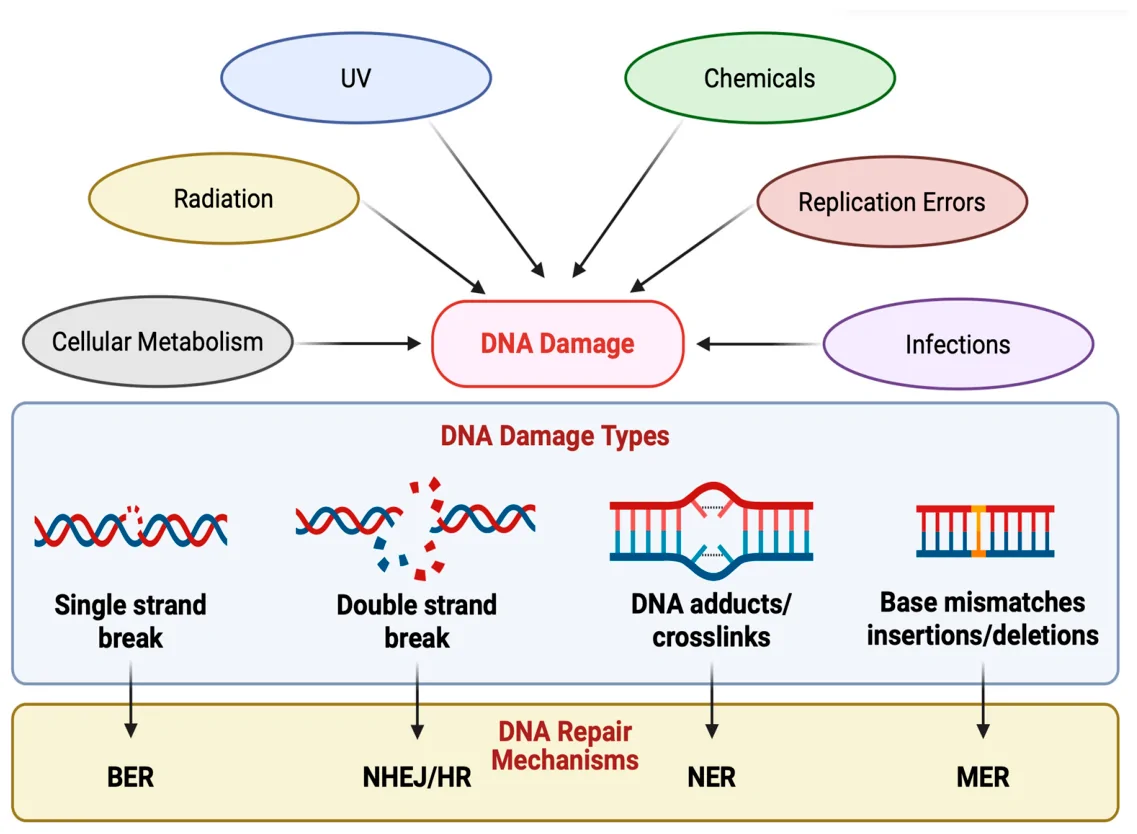
Major causes, types, and repair mechanisms of DNA damage. This figure illustrates the principal sources of DNA damage including UV radiation, chemicals, replication errors, infections, cellular metabolism, and other forms of radiation, which can compromise genome integrity. The resulting DNA damage manifests as single-strand breaks, double-strand breaks, DNA adducts/crosslinks, and base mismatches or insertions/deletions. Each type of DNA damage is addressed by specific cellular repair pathways: base excision repair (BER) primarily fixes single-strand breaks, homologous recombination (HR) and non-homologous end joining (NHEJ) repair double-strand breaks, nucleotide excision repair (NER) resolves bulky adducts and crosslinks, and mismatch repair (MMR) corrects base mismatches and small insertions/deletions. These coordinated DNA repair mechanisms are essential for maintaining genomic stability and preventing mutations that could lead to diseases such as cancer. Created using https://biorender.com/ (the figure is licensed under CC BY 4.0).

DNA damage caused by various stress factors must be efficiently repaired to restore normal cellular function and maintain genomic stability [21]. The presence of DNA lesions activates the DNA damage response (DDR), a cellular mechanism that detects and rectifies errors, often eliminating cells with unrepaired damage via apoptosis to preserve tissue homeostasis. DDR triggers the activation of multiple DNA repair pathways, each tailored to specific types of damage. Key repair mechanisms include base excision repair (BER), nucleotide excision repair (NER), homologous recombination (HR), non-homologous end joining (NHEJ), and mismatch repair (MMR), with each mechanism corresponding to distinct DNA lesions [22] [Figure 1].

SSBs are breaks in the phosphodiester backbone of one strand of the DNA double helix that may be formed by the damage of the deoxyribose sugar of DNA by ROS, by collision of DNA or RNA polymerases with the cleavage complex formed as an intermediate during DNA topoisomerase 1 (TOP1) activity, or as an intermediate in various DNA excision repair pathways [23,24]. The PARP1 family of nuclear proteins plays a central role in the repair of SSBs. However, in cells harboring mutations in TSGs like BRCA1/2, the repair of double-strand breaks by the mechanism of HR is impaired, and hence they depend on PARP-mediated SSB repair pathways for survival. Inhibition of PARP in these HR-deficient cells leads to the accumulation of DNA damage, the collapse of the replication fork, and synthetic lethality, which makes PARP proteins attractive therapeutic targets in cancer treatment [15,25,26]. The role of PARP in DNA damage response was first recognized in the late 1970s from studies that demonstrated that DNA damage induced by alkylating agents led to PARP activation and cellular NAD+ depletion [27]. Thus, PARP proteins have emerged as important therapeutic targets due to their central role in DNA repair, and substantial progress has been made in the development of small-molecule PARP inhibitors over the past several decades [28].

Numerous studies have highlighted the critical role of PARP in SSB repair. PARP inhibitors have demonstrated cytotoxic efficacy in various cancers, including breast, ovarian, and prostate cancers [29,30]. Several PARP inhibitors exhibit limited selectivity and may inhibit other PARP family members, including PARP2 and PARP3 [31,32]. To date, four PARP inhibitors—olaparib, niraparib, rucaparib, and talazoparib—have received FDA approval after extensive clinical evaluation for the treatment of BRCA1/2-mutated and HR-deficient cancers [33].

Clinically, resistance to PARP inhibitors poses a major challenge, as it is associated with increased rates of disease recurrence and reduced overall survival. For example, in ovarian cancer, roughly 40–50% of patients with BRCA1/2 mutations show intrinsic resistance to PARPi, and overall resistance is observed in 40–70% of patients. This signifies the urgent need to understand resistance mechanisms and develop strategies to overcome them [34]. Mechanisms of resistance include secondary BRCA mutations, restoration of HR, increased drug efflux, and epigenetic alterations, all of which undermine the efficacy of PARPi and limit long-term patient benefit [35]. This review explores the DNA repair pathways involved in PARPi resistance and highlights emerging therapeutic strategies designed to overcome this challenge and enhance the durability of PARPi-based treatments.

## 3. Role of PARP in DNA Repair Mechanism

Single-strand DNA lesions can arise through both direct and indirect mechanisms [24]. Direct damage can be caused by external agents such as IR by the introduction of strand breaks and base modifications. Ionizing radiation can also result in SSBs indirectly by the action of ROS and RNS which are generated by the radiolysis of water [36]. SSBs also appear as intermediates during BER, when damaged bases are removed by DNA glycosylases to generate abasic sites which are then cleaved at the phosphodiester backbone [37,38]. SSBs that remain unrepaired can collide with replication forks leading to fork collapse into DSBs which activates DNA damage response pathways [24].

PARP family proteins have a well-established role in the BER pathway. The PARP family consists of 17 proteins involved in diverse cellular functions, including DNA repair, metabolism, maintenance of the telomere, and immune responses [39]. PARP1 (Poly(ADP-ribose) Polymerase 1) is the most prominent member and is a key nuclear protein actively participating in DNA repair [40,41]. Upon occurrence of an SSB, PARP1 rapidly binds to the site of DNA damage and initiates the BER process. PARP1 has three broad functional regions with six independently folded domains. One of them, the N-terminal DNA-binding domain, contains two zinc finger motifs (ZnF1 and ZnF2) that specifically recognize and bind to the SSB, inducing a conformational change that activates the enzyme [42,43]. Activated PARP1 catalyzes the cleavage of NAD+ into nicotinamide and ADP-ribose, and subsequently transfers ADP-ribose units to itself and other acceptor proteins, forming long, branched and negatively charged PAR (poly-ADP-ribose) chains through the process of PARylation [40,41,44,45].

PARylation serves as a signal to recruit DNA repair factors to the damage site. PARP1 is first released from the break point as a consequence of auto-PARylation. X-ray cross-complementing group 1 (XRCC1), a molecular scaffold, is then recruited. XRCC1 consists of three domains—an N-terminal domain (NTD), a central BRCT domain (BRCT1) and a C-terminal BRCT domain (BRCT2). The BRCT1 domain of XRCC1 binds to the PAR chain and also recruits other associated proteins called cargoes to the sites of damage. These cargoes include DNA polymerase β (Polβ), which binds to the NTD, the DNA ligase 3 (LIG3), which binds to BRCT2, and polynucleotide kinase phosphatase (PNKP), aprataxin (APTX), and aprataxin and PNKP-like factor (APLF). This coordinated action results in the generation of 5′ phosphate and 3′ hydroxyl termini, which are subsequently joined by DNA ligase III [39,41]. In cases where multiple SSBs or gaps are present, additional repair factors such as flap endonuclease 1 (FEN1) and DNA ligase I (Lig I) are recruited to process and seal the DNA ends [46]. Through these sequential steps, PARP1 and its associated proteins ensure efficient repair of SSBs, thereby maintaining genome integrity and cellular viability.

## 4. Synthetic Lethality

Genomically unstable cancer cells frequently harbor multiple mutations in DNA replication and repair genes, leading to replication stress and accumulation of DNA lesions including SSBs [47]. PARP proteins act as signal transducers for SSBs, facilitating the BER pathway [48]. If SSBs are not efficiently repaired, they result in replication fork collapse and eventually give rise to DSBs, which are typically resolved by the high-fidelity HR pathway. However, cells with BRCA1/2 mutations have severely impaired HR-mediated repair ability [49,50]. In many cancers such as breast, ovarian, prostate, and pancreatic cancers, patients may carry tumors with biallelic mutations in BRCA1 or BRCA2 or other HR deficiencies [16]. Such cells are dependent on PARP-mediated repair pathways for survival. This genetic vulnerability has driven the development of specific inhibitors targeting PARP enzymes [51]. PARPi are particularly effective against BRCA-deficient tumors, leading to selective cancer cell death while sparing normal cells [52]. Early preclinical work in BRCA1-deficient mouse mammary tumor models confirmed this selective vulnerability, showing markedly greater sensitivity to the PARP inhibitor olaparib (AZD2281) than in BRCA1-proficient tumors, both as monotherapy and in combination with platinum-based chemotherapy [53].

**Figure 2:**
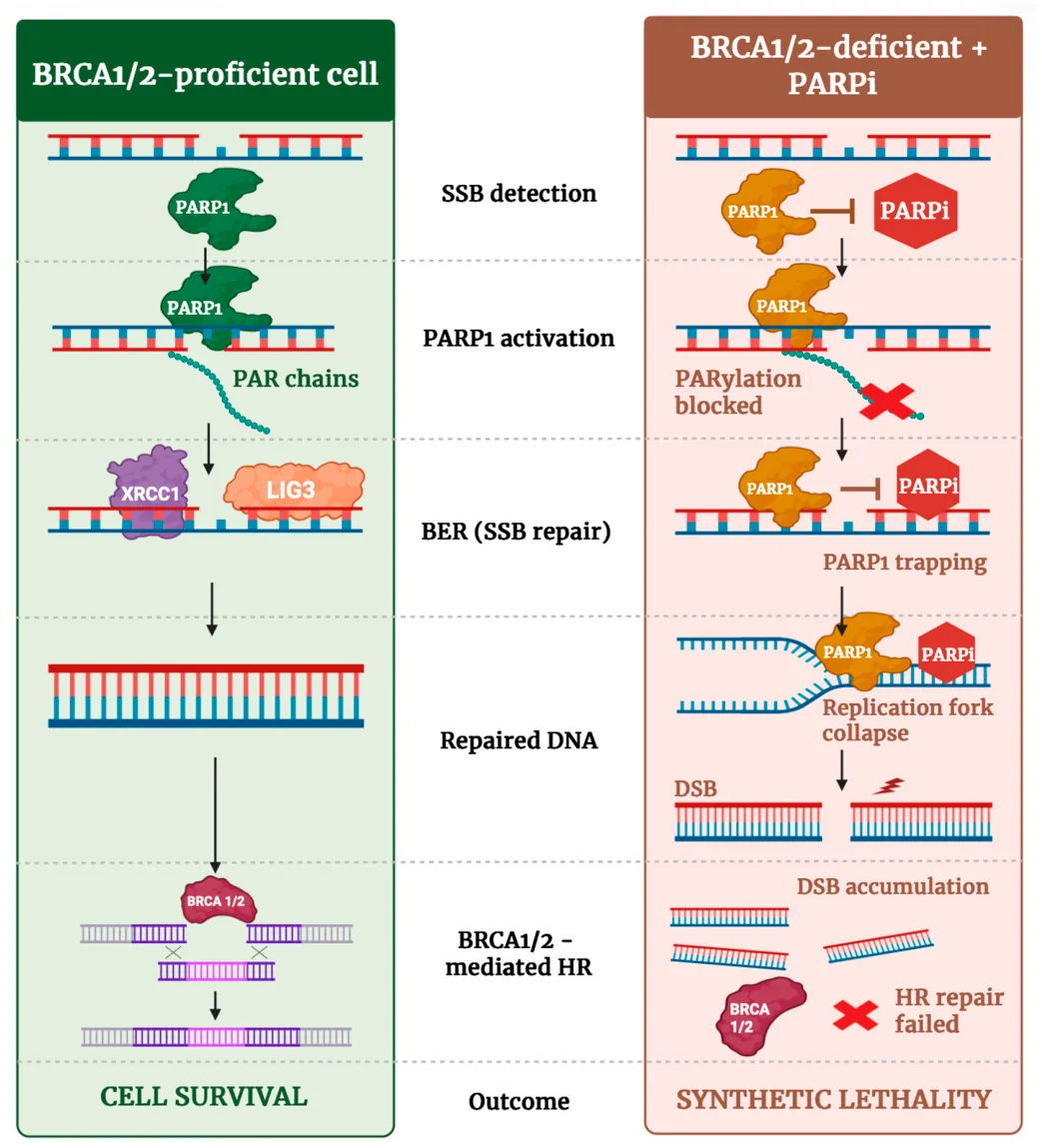
Synthetic lethality mechanism of PARP inhibitors in BRCA1/2-mutant cancers. In BRCA1/2-proficient cells, PARP1 detects SSBs, initiates BER via PARylation, and recruits XRCC1 and Ligase 3, leading to successful repair and cell survival. In BRCA1/2-mutant cells, PARP inhibitors competitively inhibit NAD+ binding, blocking PARylation and trapping PARP1 on DNA. This impedes replication fork progression, generating DSBs that cannot be resolved by homologous recombination in HR-deficient cells. Error-prone alternative repair pathways lead to genomic instability and cell death, which are the basis of synthetic lethality. Created using https://biorender.com/ (the figure is licensed under CC BY 4.0).

Synthetic lethality occurs when the simultaneous loss of two genes leads to cell death, whereas loss of any single gene alone does not affect cell viability [54]. In BRCA1/2-mutant cells, HR is impaired, making them dependent on PARP-mediated DNA damage response pathways. PARP inhibitors exploit this vulnerability by blocking PARP activity, which results in the accumulation of unrepaired DNA lesions. PARPi also trap PARP1 and PARP2 on damaged DNA, resulting in the formation of PARP-DNA complexes that are more cytotoxic than unrepaired SSBs alone, thereby promoting selective cancer cell death while relatively sparing normal cells with intact HR [16,55]. Emerging evidence indicates that PARPi-induced cytotoxicity may arise from replication-associated ssDNA gaps and replication stress and not just from unrepaired SSBs converting into DSBs [56,57] [Figure 2].

Synthetic lethality is now being applied widely as a therapeutic strategy, broadening its relevance beyond BRCA-mutant tumors to include cancers harboring a range of DNA repair pathway defects [58]. Ongoing research is expanding the repertoire of synthetic lethal interactions, paving the way for novel combination therapies and personalized cancer treatments.

In HR-deficient cells, some of the breaks caused by PARP inhibitors are repaired through error-prone end joining, particularly MMEJ. As discussed later, this pathway can contribute to resistance but may also serve as a therapeutic target [59,60].

## 5. PARP Inhibitors in DNA Repair

PARP proteins are key mediators of DNA repair, binding to damaged DNA primarily via the N-terminal zinc finger domains Zn1 and Zn2, and catalyzing PARylation using NAD+ [42]. This process recruits DNA repair factors and facilitates the resealing of DNA breaks. In rapidly dividing cancer cells, frequent DNA lesions are often managed by PARP-dependent repair mechanisms [42]. The development of PARPi has revolutionized cancer therapy, particularly for patients with germline BRCA1/2 mutations. BRCA1/2 are TSGs essential for HR mediated DSB repair; mutations in these genes lead to a dependency on PARP-mediated SSB repair which results in synthetic lethality when combined with PARPi treatment [42]. However, resistance to PARPi remains a significant clinical challenge—more than 40% of BRCA1/2-deficient patients exhibit intrinsic resistance, and even the majority of initially responsive patients acquire resistance over time [34]. Once resistance develops, the effectiveness of these drugs diminishes, increasing the risk of cancer recurrence and reduced overall survival [35].

### 5.1. Mechanism of Action of PARP Inhibitor in DNA Repair

**Figure 3:**
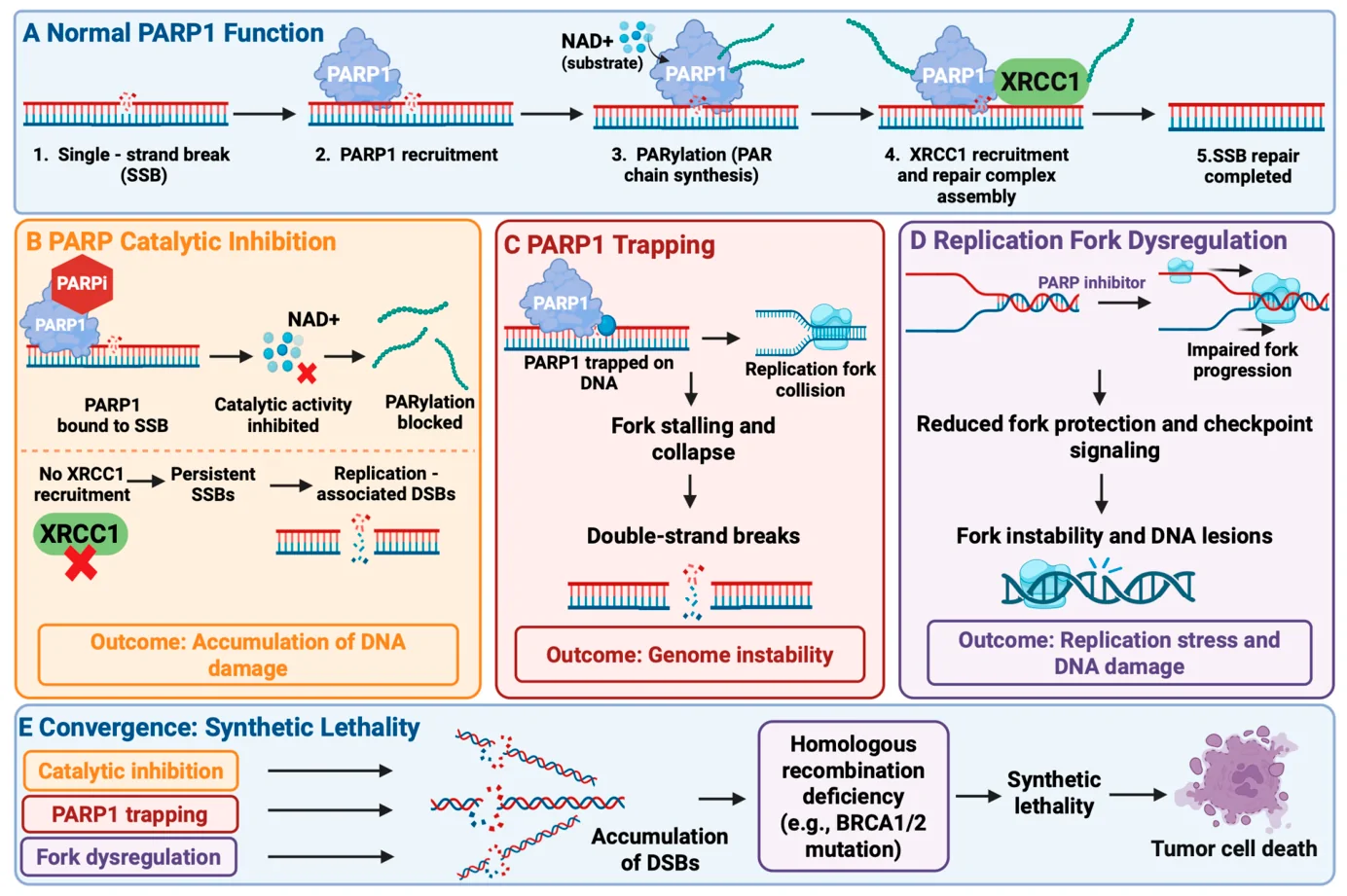
Mechanism of Action of PARP Inhibitors (PARPi). (**A**) Normal PARP1 function in SSB repair via PARylation-mediated recruitment of the BER complex. (**B**) PARPi-mediated catalytic inhibition blocks PARylation, preventing XRCC1 recruitment and causing replication-associated DSB accumulation. (**C**) PARP1 trapping at repair intermediates leads to replication fork collapse and genome instability. (**D**) PARPi-induced replication fork dysregulation causes replication stress and DNA lesions. (**E**) Convergence of these mechanisms drives DSB accumulation that cannot be resolved in HR-deficient (BRCA1/2-mutant) cells, resulting in synthetic lethality and tumor cell death. Created using https://biorender.com/ (the figure is licensed under CC BY 4.0).

PARP proteins, particularly PARP1, are essential for initiating the BER pathway in response to SSBs. Inhibition of PARP has emerged as a powerful strategy to suppress cancer cell proliferation, especially in tumors with HR deficiencies, such as BRCA1/2 mutations. PARPi are now used as frontline therapies [61] and in combination regimens to reduce disease recurrence, although their effectiveness in reducing disease recurrence may vary across patient populations.By exploiting synthetic lethality, PARPi selectively induce cytotoxicity in HR-deficient tumors and can also enhance the effects of chemotherapy and radiation [Figure 3] [62].

#### 5.1.1. Enzymatic Inhibition of PARP

PARP inhibitors competitively bind to the NAD+ catalytic site of PARP1, preventing the enzyme from utilizing NAD+ to form Poly(ADP-ribose) (PAR) chains—a critical step for initiating BER [61,63]. This blockade suppresses the formation of PAR chains, thereby inhibiting the recruitment of DNA repair proteins and stalling the SSB repair process [64]. During SSB repair, once PAR chain formation has occurred and XRCC1 is recruited, removal of PAR chains is essential for repair completion. Poly(ADP-ribose) glycohydrolase (PARG) plays a pivotal role throughout this process by cleaving PAR chains through both exoglycosidic and endoglycosidic activities, facilitating PARP1 dissociation from DNA and enabling repair to proceed. If PAR chains accumulate excessively, this can trigger cell death via apoptosis or a specialized form of cell death known as parthanatos [65].

The most important effect depends on the genetic background of the cells. One recent review suggests that, in BRCA-deficient cells, the loss of PARP1 catalytic activity may be more important for cell death than PARP trapping [66]. Trapping may play a larger role in other DNA repair-defective cells. A 2026 review also highlights that PARP inhibitor-induced cell death cannot be explained by a single mechanism. Catalytic inhibition, PARP trapping, gap accumulation, replication stress and conflicts between transcription and replication can all contribute with their relative importance depending on the inhibitor, BRCA status and cell type [19]. These differences also matter for resistance as reduced PARP trapping is one way cancer cells can become less sensitive to PARP inhibitors [67].

#### 5.1.2. PARP Trapping

Beyond catalytic inhibition, many PARP inhibitors induce a phenomenon known as PARP trapping. Live-cell imaging studies show that PARP1 is retained at DNA damage sites by two independent mechanisms: catalytic inhibition, which causes repeated ineffective cycles of binding and dissociation of PARP1, and allosteric modulation by specific PARPi which increases the duration of the bound state of PARP1 [68]. The precise contributions of each mechanism remain a subject of ongoing debate [64]. This results in the “trapping” of PARP1 on chromatin at sites of DNA damage, especially when combined with DNA-damaging agents like temozolomide or methyl methanesulfonate (MMS) [63,69]. Trapped PARP1-DNA complexes are highly cytotoxic, as they block DNA replication and transcription, leading to replication fork stalling, fork collapse, and the formation of DSBs [69]. This mechanism is particularly lethal in HR-deficient cells, which cannot efficiently repair DSBs. PARP trapping varies between inhibitors and is linked to how long the drug remains bound to PARP1 [64]. A 2025 study found that combining olaparib with the cytosine analogue 5-hydroxymethyl-2′-deoxycytidine (5hmC) increased PARP1 trapping in BRCA1/2-deficient cells. This combination also increased replication gaps and γH2AX foci and restored olaparib sensitivity in resistant cells where resistance was caused by 53BP1 loss or increased fork protection [70]. However, this approach may not work when resistance is caused by BRCA reversion. Since the study used engineered cell lines, further research is needed to find out whether the findings will be applicable to patients [70].

Clinically approved PARPi traps not only PARP1 but also PARP2. These PARPi trap both paralogs at sites of DNA damage and trapping potency varies markedly between inhibitors [71]. PARP2 trapping occurs through a WGR-domain-mediated physical stalling mechanism, distinct from the PARP1- and PAR-dependent rapid exchange that governs PARP2 recruitment under normal conditions [72]. Additionally, certain clinical PARPi retain PARP2 on DNA breaks through an allosteric mechanism involving catalytic-to-DNA-binding domain communication [73]. Critically, PARP2 inhibition and trapping, rather than PARP1, has been attributed as the principal driver of hematological toxicities seen clinically with dual PARP1/2 inhibitors, given PARP2’s essential role in hematopoietic progenitor survival [73,74]. This distinction has motivated next-generation PARP1-selective inhibitors such as AZD5305, which achieves approximately 500-fold selectivity for PARP1 over PARP2, retaining antitumor efficacy while markedly reducing hematotoxicity in preclinical models [74]. It achieves this by binding itself to the NAD^+^ catalytic site present in the structure of PARP1 and preventing auto-PARylation from occurring, and makes PARP1 stay bound to the DNA single-stranded breaks, resulting in the breakdown of the replication fork and double-strand DNA damage [75]. Therefore, cancer cells struggling with impaired HR repair mechanisms consisting of mutations in the BRCA1/2 genes are eliminated via synthetic lethality, yet this leaves the process of PARP2-dependent functions [76]. The phase I/II trial (NCT04644068) has clinically demonstrated the efficacy of AZD5305 on patients suffering from advanced HR-deficient solid tumors, where response rates of ~36–48% were reported in heavily pretreated breast and ovarian cancer cohorts. The study was designed as a multi-center first-in-human trial involving monotherapy as well as combination therapy on patients exhibiting mutations in BRCA1/2, PALB2, RAD51C, or RAD51D [77]. Currently active Phase III clinical trials include EvoPAR-PR01 (NCT06120491) for treatment of metastatic castration-sensitive prostate cancer [78] and EvoPAR-Breast01 (NCT06380751) for treatment of breast cancer with BRCA/PALB2 mutations [75]. The benefits of AZD5305 include lower myelosuppression, better tolerability, and continuous efficacy in treatment, but the drawbacks include the threat of a development of resistance against PARP inhibitors (due to BRCA reversion mutations), lack of long-term safety data, and currently limited mature clinical data from Phase III trials [77].

#### 5.1.3. Impact on Replication Fork Progression

PARP inhibitors also influence DNA replication dynamics. When PARPi binds to the NAD+ active site of PARP1, PARylation is blocked, leading to replicative stress. Recent studies show that the effect of PARPi on the replication fork includes fork acceleration followed by a secondary reduction in origin firing [79,80]. The molecular basis of this acceleration has now been identified: both POLA1 (DNA polymerase α) and PRIMPOL act as mediators of PARPi-induced fork acceleration, and both the polymerase and primase activities of the POLA complex are required for this phenomenon [80]. The mechanistic explanation is that PARylation and p21 are suppressors of fork speed in a coordinated network regulated by PARP1 and p53. PARPi disrupts this suppression, resulting in fork acceleration [79]. DNA lesions are normally recognized and rectified at the replication fork, but due to PARP inhibition, they may remain undetected by the replication machinery. This would lead to the accumulation of ssDNA gaps associated with replication [79,80]. These gaps can arise in two different ways: PRIMPOL-mediated repriming on the leading strand, and defective maturation of the lagging strand due to impaired Okazaki fragment processing [50,56]. In BRCA1/2-deficient cells, which lack replication gap suppression (RGS), PARPi treatment accelerates fork progression and increases fork asymmetry, resulting in defective DNA replication during the S phase. Enhanced fork progression and reduced fork stalling decrease the opportunity for repair, leading to the propagation of mutations and genomic instability [79,80]. The synthetic lethality between PARPi and BRCA1/2 deficiency thus drives the accumulation of irreparable DNA damage and cancer cell death. Proteins involved in regulating origin firing, including MTBP, and Treslin, also affect fork speed. Depletion of MTBP or Treslin raises the fork speed beyond the threshold compatible with genome stability and induces a DNA damage response, further demonstrating that dysregulated fork speed is a broad trigger of replication stress [79]. The severity of these effects is dose-dependent. Higher concentrations of PARPi induce more fork acceleration and greater fork asymmetry [79].

The way in which cells process these gaps also affects how much damage they accumulate. Using DNA fiber analysis, electron microscopy and biochemical assays, Seppa et al. found that MRN–CtIP, EXO1 and DNA2–WRN/BLM resect single-stranded DNA gaps from both ends in PARPi-treated cells. In BRCA1-deficient cells, this process produced larger gaps (median 177 nt, compared with 120 nt in BRCA1-proficient cells). These gaps remained unrepaired and became DNA breaks during the next round of replication when they encountered advancing replication forks [81]. This suggests that faster replication fork progression may not be the main cause of PARPi toxicity. Although POLA1 and PRIMPOL are required for PARPi-induced fork acceleration, depleting POLA1 actually increases gap formation and makes cells more sensitive to PARP inhibitors. This supports the idea that DNA gaps can contribute to PARPi toxicity independently of fork speed [56,80].

Evidence for the role of DNA gaps in PARP inhibitor response is growing. Recent studies have shown that how gaps are processed by nucleases can affect whether they are repaired in BRCA1-deficient cells [81]. Gap suppression may also contribute to resistance. Loss of nuclear DNA ligase III restored ssDNA gaps and reversed PARPi resistance in BRCA1/53BP1 double-deficient cells. Meanwhile, LIG3 depletion improved PARPi efficacy in mouse mammary tumors that lacked BRCA1 [82]. Gap accumulation may also be a potential biomarker as it was associated with sensitivity to a USP1 inhibitor in patient-derived ovarian tumor organoids [83]. However, most of this evidence comes from cell lines, organoids and xenograft models and only five patient-derived organoids were examined. The DNA fiber assay used to measure gaps is technically demanding and may produce variable results between laboratories [83]. In addition, the organoid findings show an association between gap accumulation and drug sensitivity, but do not establish that the gaps directly cause cell death. Further studies are therefore needed before gap measurements can be used as a reliable clinical biomarker.

MMEJ may also repair some of the breaks that arise later. A 2026 study found that PARP1/2 inhibition increased Polθ-dependent MMEJ at I-SceI-induced double-strand breaks in repair-proficient cells, and that this effect depended on HR [84]. However, the study did not examine whether the same occurs at replication-associated breaks following PARP inhibitor treatment. This possibility is discussed further below in the context of MMEJ and POLQ as potential therapeutic targets.

## 6. Mechanism of PARP Inhibitor Resistance

Despite the initial effectiveness of PARPi such as olaparib, many patients develop resistance after prolonged treatment. This resistance arises through several molecular mechanisms, each undermining the cytotoxic effects of PARPi and contributing to tumor relapse. Key resistance mechanisms include.

### 6.1. Loss of PARP1 Expression

PARP1 is essential for PARPi-induced synthetic lethality. In tumor cells without BRCA1/2 mutations, loss of PARP1 expression can grant resistance by removing the target of PARPi-mediated trapping, thereby preventing the accumulation of cytotoxic PARP1-DNA complexes [18]. On the other hand, in cells with BRCA1/2 mutations, PARP1 loss leads to synthetic lethality and therefore cannot act as a resistance mechanism in the PARPi target population [18,85]. PARP1 expression may be lost in tumor cells due to various mechanisms such as deletion of the PARP1 gene or mutations that deactivate PARP1. In addition, epigenetic modifications such as hypermethylation of promoters may cause PARP1 silencing, and repression occurring at the transcriptional level and regulation at the post-transcriptional level could play a role as well. All these processes contribute to lower or absent levels of PARP1, thus inhibiting the formation of lethal complexes between PARP1 and DNA. On the other hand, cells without BRCA1/2 genes need PARP1 protein for PARP inhibitors to work efficiently, and therefore, in case of complete PARP1 loss, cells will no longer be sensitive to PARP1 inhibitors [18,25,28,71].

### 6.2. Drug Efflux

Increased ABCB1 and ABCG2 expression confers PARPi resistance by actively exporting the drug from cells. ABCB1 upregulation drives olaparib and rucaparib resistance in ovarian cancer cells, an effect which can be reversed using ABCB1 inhibitors [86]. Rucaparib is also a direct transport substrate of both ABCB1 and ABCG2, with combined loss of both transporters increasing drug bioavailability roughly four-fold in mice [87]. Elevated ABCB1 and ABCG2 expression leads to PARP inhibitor resistance via ATP-dependent drug efflux, which decreases intracellular PARPi levels and PARP1 retention on the DNA [86]. The substrates of these transporters include olaparib and rucaparib, and high levels of the transporters reduce the clinical efficacy [86,87]. Studies using ABCB1-inhibiting drugs like tariquidar, elacridar, or verapamil have shown the potential of restoring intracellular PARPi accumulation and resensitizing resistant ovarian cancer models [86]. Double inhibition or loss of ABCB1 and ABCG2 raises brain-to-plasma drug distribution for PARP inhibitors such as rucaparib and olaparib, though systemic exposure is comparatively less affected [88].

### 6.3. Disruption of PARG Activity

Under normal physiological conditions, PARG is crucial for removing PAR chains after DNA repair. This facilitates the dissociation of PARP1 from DNA, completing SSB repair. However, under PARPi treatment, PARP1 continues to make low levels of PAR. Ordinarily, PARG rapidly degrades the residual PAR, fortifying the suppression of DNA repair signaling. However, when PARG is lost or inactivated, the residual PAR is able to restore downstream repair factor recruitment, reducing the effectiveness of PARPi [89]. Studies in BRCA2^−/−^ Trp53^−/−^ murine models have shown that PARG loss confers PARPi resistance, independent of drug efflux or HR restoration [90]. Loss of PARG reduces the accumulation of toxic PARP1-DNA complexes and restores controlled replication fork progression [90,91,92].

## 7. Replication Fork Stabilization

### Fork Protection Mechanisms

**Figure 4:**
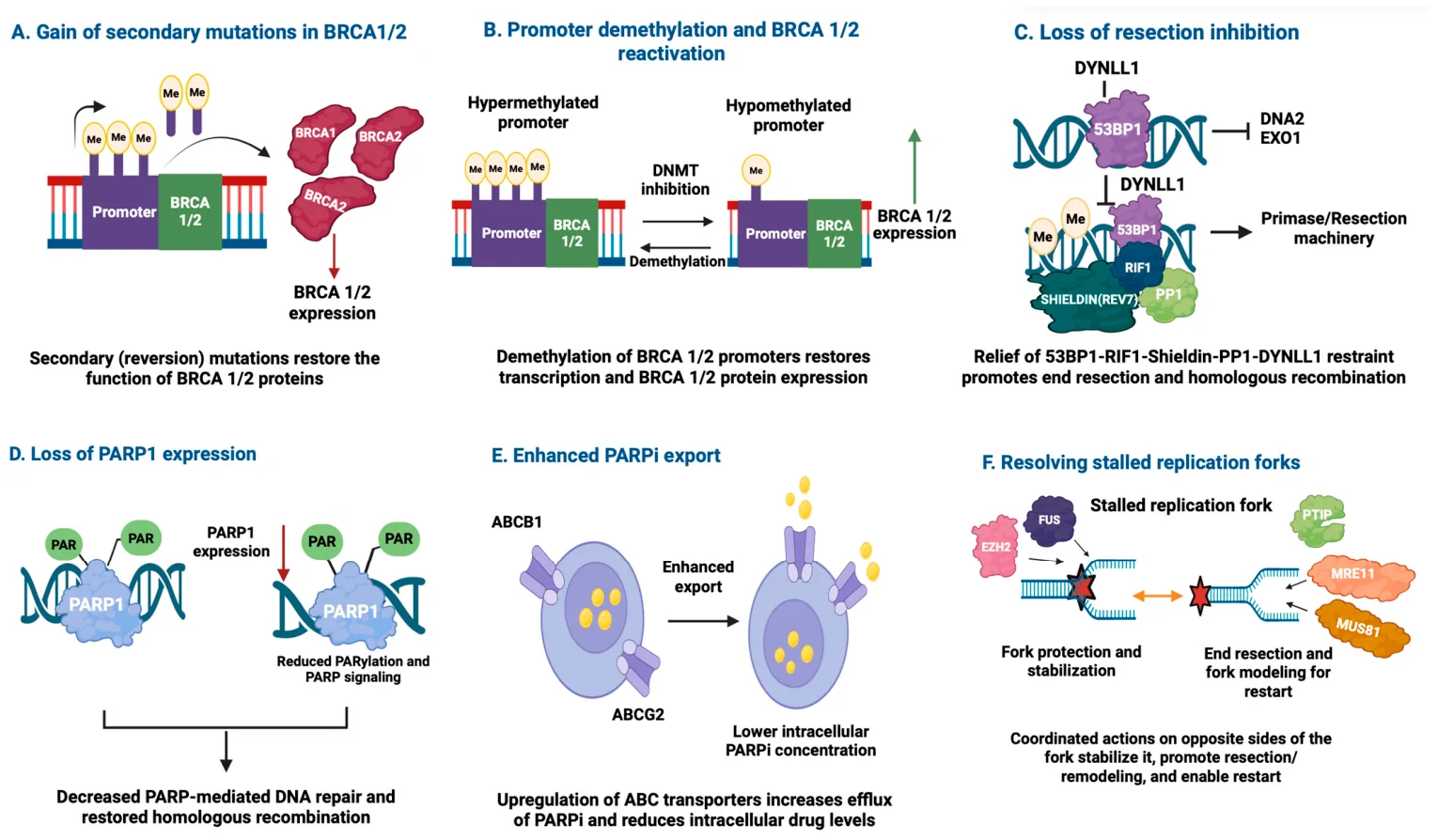
Mechanisms of BRCA1/2 Reactivation and PARP Inhibitor Resistance in Cancer Cells. This figure depicts molecular mechanisms that restore BRCA1/2 function and modulate DNA repair, summarizing key pathways by which cancer cells acquire resistance to PARP inhibitor (PARPi) therapy. (**A**) Gain of secondary mutations in BRCA1/2 genes can lead to the production of functional BRCA proteins, restoring DNA repair capacity. (**B**) Demethylation of BRCA1/2 promoters reverses gene silencing, allowing BRCA1/2 expression and functional protein production. (**C**) Loss of resection inhibition, such as through DYNLL1-mediated regulation of 53BP1, facilitates DNA end resection and repair by nucleases like DNA2 and EXO1. (**D**) Loss of PARP1 expression leads to restoration of PARP signaling through dePARylation or PAR accumulation. (**E**) Enhanced PARPi export via ABCB genes and MDR/P-gp efflux pumps reduces intracellular PARPi concentration. (**F**) Resolution of replication fork stalling, which can also contribute to therapeutic resistance. These mechanisms collectively interfere with the therapeutic impact of PARP inhibitors. Created using https://biorender.com/ (the figure is licensed under CC BY 4.0).

BRCA1/2 play a crucial role in protecting the replication fork, especially during fork reversal and stabilization under replication stress. In BRCA-deficient cells, nucleases such as MRE11 and MUS81 degrade stalled forks, leading to fork collapse and chromosomal aberrations [93,94,95]. However, resistance can arise through stabilization of replication forks, either by loss of nucleases or by upregulation of fork protection factors (e.g., loss of RADX, an antagonist of RAD51, can restore RAD51-mediated fork protection and contribute to PARPi resistance). The role of MUS81 in fork stability is context-dependent and may involve both fork collapse and stabilization [96,97]. EZH2 deposits H3K27 trimethylation at stalled forks and recruits the nuclease MUS81, while PTIP deposits H3K4 methylation to recruit MRE11. Loss of either of these proteins removes the corresponding nuclease and thereby stabilizes the fork and confers PARPi resistance [93]. PTIP loss confers resistance in both BRCA1- and BRCA2-deficient cells, whereas EZH2 loss confers resistance only in BRCA2-deficient cells, indicating these are two separable pathways [89]. Additionally, depletion of chromatin remodelers such as SMARCAL1, ZRANB3, and HLTF, which participate in fork remodeling and MRE11-mediated degradation, can also promote PARPi resistance [Figure 4A–C] [98,99].

## 8. Restoration of DNA Repair to Eliminate DSBs: DNA Damage Response in PARPi Resistance

### 8.1. Restoration of HR

HR is the primary error-free DNA repair pathway in eukaryotic cells, relying on BRCA1/2 for accurate repair of DSBs using the sister chromatid as a template. In many breast and ovarian cancers, BRCA1/2 loss leads to HR deficiency, making cells reliant on error-prone pathways like NHEJ or MMEJ. Treatment with PARPi exploits this vulnerability, but resistance often develops through restoration of HR. When a tumor restores the function of the BRCA1/2 or PALB2 genes, HR comes back and the breaks caused by PARP trapping get repaired. A secondary mutation that restores the reading frame lets RAD51 load onto resected DNA ends again [100,101]. In the TOPARP-B prostate cancer cohort, reversions were found in 79% of BRCA2/PALB2-mutated tumors by the end of treatment [102].

HR restoration in PARPi-resistant tumors can occur via three main mechanisms.

#### 8.1.1. Secondary Mutations in BRCA1/2

Secondary or “reversion” mutations in BRCA1/2 can restore the open reading frame and gene function, reactivating HR. This genetic restoration allows tumor cells to repair DSBs efficiently, reducing PARPi efficacy. Such reversion mutations are frequently observed in breast and ovarian cancers following PARPi or platinum-based chemotherapy [100,101]. Similar mechanisms have been identified in other HR-related genes, including *RAD51C*, *RAD51D*, and *PALB2*.

#### 8.1.2. Demethylation of BRCA1/2 Promoters

Epigenetic silencing of BRCA1 and less commonly BRCA2 via promoter hypermethylation can be reversed, leading to gene re-expression and HR restoration [103,104]. Demethylation or gene fusion events can reactivate BRCA1, enabling resistance to PARPi and platinum drugs. Studies in patient-derived xenograft (PDX) models have shown that loss of BRCA1 promoter hypermethylation or de novo gene fusions can restore BRCA1 expression and confer drug resistance [105].

#### 8.1.3. Loss of Resection Inhibition

In BRCA1-deficient cells, the DNA end-resection process necessary for HR is normally suppressed by 53BP1 and its downstream effectors, including RIF1, REV7, and the Shieldin complex (composed of SHLD1, SHLD2, SHLD3, and REV7). The Shieldin complex acts as a critical downstream effector of 53BP1, protecting DNA ends from excessive resection and thereby enforcing HR deficiency. Loss of any component in this pathway such as 53BP1, RIF1, REV7, or Shieldin removes this block, allowing DNA end resection to proceed and partially restoring HR even in the absence of functional BRCA1, thus leading to PARPi resistance. Similarly, DYNLL1 regulates end resection by preventing EXO1 and DNA2 binding and by mediating 53BP1 dimerization, which activates downstream factors including RIF1, REV7, Shieldin, the CST complex, and Pol α to fill in single-stranded DNA gaps. Disruption of these pathways promotes end resection and HR restoration, driving resistance to PARPi [106]. Loss of 53BP1 enables HR restoration specifically in BRCA1-deficient, but not BRCA2-deficient, cells. Clinical resistance to PARPi can also arise from secondary or reversion mutations in BRCA1/2, which restore the open reading frame and functional protein expression, reactivating HR and conferring cross-resistance to platinum agents and PARPi [107]. Together, these genetic and epigenetic alterations signify the complexity of HR restoration as a central mechanism of PARPi resistance in cancer [Figure 4D–F] [106]. In BRCA1-deficient cells, 53BP1 and its effectors RIF1, REV7 and Shieldin keep DNA ends from being resected, which pushes breaks toward NHEJ. When any of these is lost, resection resumes and RAD51 can load without BRCA1, so HR is partly restored and the cells tolerate PARPi [89,108]. This works for BRCA1 but not BRCA2 deficiency, because the loading step in BRCA2-null cells still needs BRCA2 [19,89].

These three mechanisms differ in how much clinical evidence supports them, how often they occur and whether they can be targeted. BRCA1/2 reversion mutations have the strongest clinical evidence. In a ctDNA study of 47 patients with metastatic breast cancer carrying BRCA1, BRCA2 or PALB2 mutations, reversions were detected in 60% of patients at progression and were the most common resistance mechanism. In some cases, they appeared in plasma before clinical progression [109]. However, their reported frequency varies across studies, with a pooled analysis of 327 patients mostly with ovarian cancer reporting a rate of about 26% [110]. Once a reversion restores the BRCA reading frame, there is currently no established way to reverse it pharmacologically (Table 1). Loss of resection inhibition, involving proteins such as 53BP1, RIF1 and Shieldin, was less common in the same breast cancer cohort and sometimes occurred alongside reversion mutations [109]. This mechanism is more relevant to BRCA1-deficient than BRCA2-deficient tumors [19]. This may create a dependency on Polθ, although this remains a preclinical therapeutic approach [60]. Promoter demethylation could potentially be reversed, but evidence for this mechanism is more limited and comes largely from patient-derived xenograft (PDX) models [8]. Recent reviews also describe it as predominantly preclinical [19]. These mechanisms can overlap within the same tumor, and the methods used to detect them are not yet standardized. Further clinical validation is therefore needed before any of the three can reliably be used to guide treatment decisions.

MMEJ may also play a role in restoring HR function. In a pooled analysis of 327 patients, deletions that restored the BRCA reading frame often showed microhomology at their breakpoints, suggesting a role for end joining [110]. However, this pattern was not seen in all cases, indicating that other repair pathways may also contribute [111].

**Table 1:** Comparative overview of PARP inhibitor resistance mechanisms. Mechanisms are compared by clinical evidence level, relationship to other mechanisms, associated biomarker and therapeutic targetability. HR, homologous recombination; ctDNA, circulating tumor DNA; IHC, immunohistochemistry; PDX, patient-derived xenograft.

Mechanism	Clinical Evidence Level	Relationship to Other Mechanisms	Associated Biomarker	Therapeutic Targetability	Reference(s)
HR restoration via BRCA1/2 reversion mutations	High—directly documented in patient tumor biopsies and ctDNA from relapsed ovarian and breast cancer cohorts	Largely independent of fork protection and drug efflux mechanisms	Tumor/ctDNA sequencing for reversion mutations; restored RAD51 function	Not currently druggable; no small-molecule strategy exists to reverse restoration of the open reading frame	[100,101]
Loss of resection inhibition (53BP1/Shieldin complex)	Moderate—validated in BRCA1-deficient patient-derived xenograft and cell line models; not yet routinely assayed in patients	Mechanistically overlapping with HR restoration, as both re-enable end resection and RAD51 loading	Shieldin complex component expression loss; RAD51 foci formation assay	Not currently druggable; a loss-of-function event with no available pharmacological reversal	[106,108]
Replication fork protection (RAD51, FANCD2, RADX, SMARCAL1/ZRANB3/HLTF)	Moderate—supported by BRCA-deficient cell line and patient-derived xenograft models	Independent of HR restoration; can co-occur with PARG loss, as both converge on fork stability	No validated clinical biomarker yet established; RAD51 foci intensity under active investigation	Targetable. ATR inhibitors and agents against the MUS81/EZH2 axis are in active development	[93,94,98,112,113]
PARG loss or inactivation	Low—demonstrated only in murine BRCA2^−/−^; Trp53^−/−^ models to date	Can co-occur with fork protection mechanisms, given shared downstream effects on fork stability	No clinical biomarker currently established	Preclinically promising; no PARG-directed clinical agents are yet available	[90]
Loss of PARP1 expression	Low–Moderate—PARP1-inactivating point mutations identified via genome-wide CRISPR-Cas9 screens in cell lines; not yet systematically documented in patient tumor biopsies	Restricted to BRCA1/2-wild-type tumors; in BRCA1/2-mutant cells, PARP1 loss instead produces synthetic lethality rather than resistance, so this mechanism cannot co-occur with BRCA reversion in the same tumor	PARP1 gene deletion, inactivating mutation, or promoter hypermethylation (loss of PARP1 protein by IHC)	Not currently druggable; no strategy exists to pharmacologically restore PARP1 expression	[18,25,28,71]
Drug efflux (ABCB1/ABCG2 upregulation)	Moderate—demonstrated in resistant ovarian cancer cell lines and reversible with efflux inhibitors	Independent of HR- and fork-related mechanisms; a pharmacokinetic rather than DNA-repair-based resistance mechanism	ABCB1/ABCG2 transcript or protein overexpression by qPCR or immunohistochemistry	Targetable. ABCB1 inhibitors (tariquidar, elacridar, verapamil) restore intracellular PARPi accumulation preclinically	[86,87]

None of these mechanisms has been demonstrated to be strictly mutually exclusive in patient tumors; HR restoration and fork protection appear largely independent, while PARG loss and fork protection can co-occur given their shared convergence on replication fork stability.

### 8.2. Replication Fork Protection

Enhanced protection of replication forks has emerged as a PARPi resistance mechanism independent of HR restoration. RAD51 plays a dual role at replication forks, promoting fork reversal and protecting nascent DNA from nucleolytic degradation. Importantly, higher levels of RAD51 are required for fork protection than for fork reversal, explaining why some RAD51-dependent functions can be retained despite partial deficiency [112]. FANCD2 is essential for maintaining fork stability, particularly during replication stress, with FANCD2-deficient cells showing increased sensitivity to PARP inhibition due to compromised fork stability [113]. RADX acts as a negative regulator of RAD51 at replication forks, and its depletion can restore fork protection in cells deficient for BRCA1/2 or FANCD2 [112]. Chromatin remodelers like SMARCAL1, ZRANB3, and HLTF facilitate fork remodeling and protection [98]. Recent evidence indicates that SMARCA4 deficiency exacerbates replication stress upon combined ATR and PARP inhibition [114], suggesting therapeutic opportunities through targeting compensatory fork protection pathways.

### 8.3. Convergence and Clinical Relevance of Resistance Mechanisms

Despite their mechanistic diversity, these resistance pathways converge on a common endpoint. They prevent the conversion of PARP1-trapped lesions into unrepaired, lethal double-strand breaks, but they differ sharply in clinical validation. BRCA reversion mutations are currently the only mechanism that is routinely detected in patient samples, while fork protection and PARG loss remain preclinical observations awaiting clinical biomarker development.

## 9. Heterogeneity of PARPi Resistance Across Cancer Types

PARPi resistance varies across cancer types and molecular backgrounds. HR deficiency is found in about half of ovarian cancers compared with approximately 21% of breast cancers, 10% of prostate cancers and 15% of pancreatic cancers [115]. The specific gene involved also matters. PALB2 alterations show the most consistent benefit from PARP inhibitors across tumor types, while RAD51C and RAD51D alterations are clinically relevant mainly in ovarian cancer. Evidence for benefit in tumors with ATM, CHEK2 or CDK12 alterations remains limited or inconsistent [115].

The frequency of BRCA reversion mutations also differs between studies. One pooled analysis, which included mostly ovarian cancer cases, reported a reversion rate of around 26% [110] compared with 60% in a breast cancer ctDNA cohort [109]. These estimates should be interpreted cautiously, as the studies differed in their methods and prior treatments of patients. Resistance mechanisms are also influenced by disease stage, previous exposure to platinum-based chemotherapy or PARP inhibitors and hormone signaling. The same tumor may also have more than one mechanism [19,109].

Much of the mechanistic evidence comes from BRCA-mutant models. The findings therefore cannot automatically be applied to other cancer types or tumors with defects in other HR genes.

## 10. Microhomology-Mediated End Joining (MMEJ)

HR restoration, fork protection and DNA gap repair all influence how much damage accumulates in the cell. When HR is lost, cells may rely more on MMEJ to repair the resulting breaks, although this repair is error-prone.

**Figure 5:**
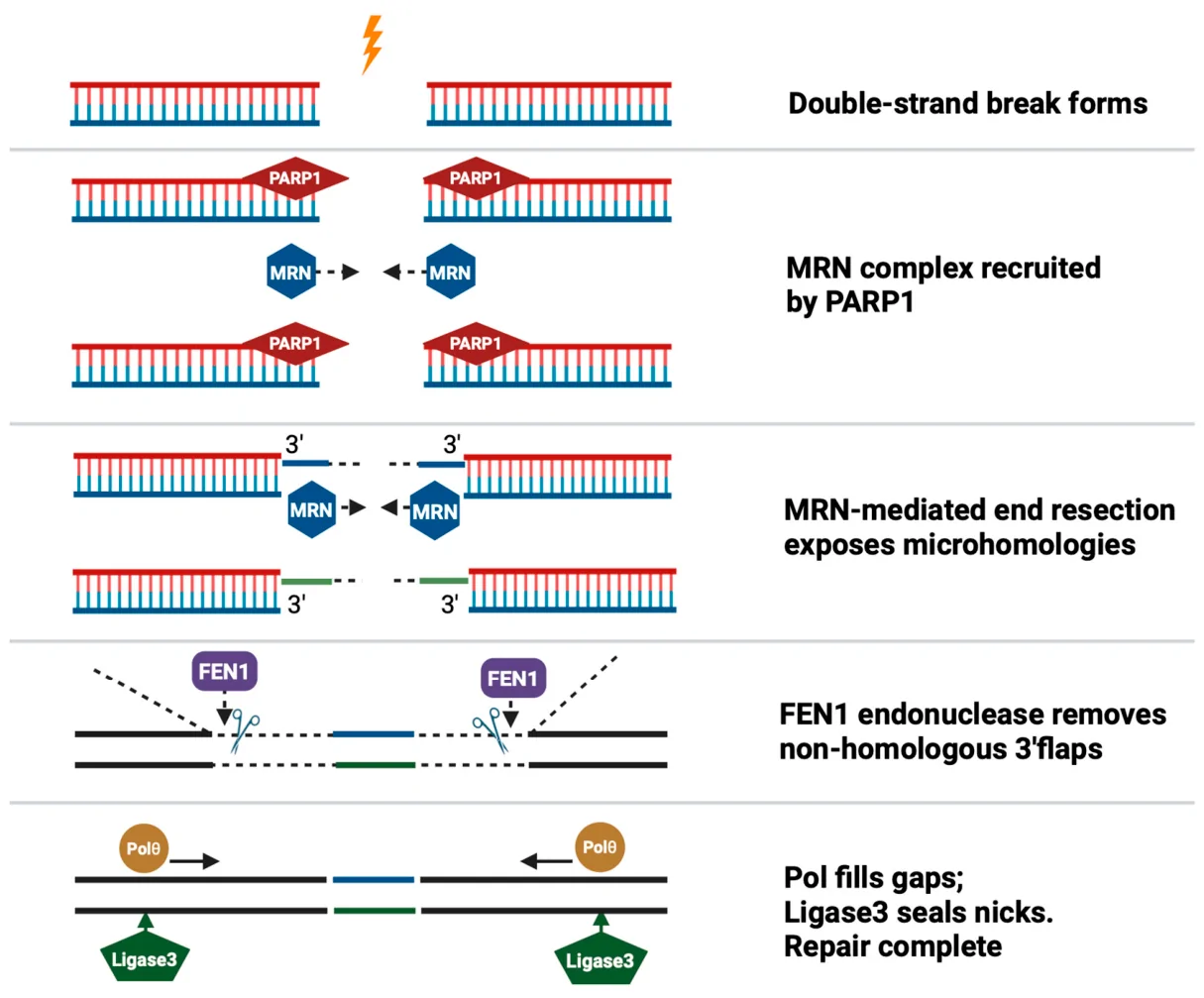
Overview of microhomology-mediated end joining (MMEJ) mechanism. This outlines the key steps in the MMEJ pathway for double-strand break (DSB) repair. (1) DSB formation exposes microhomologies of 2–20 base pairs. (2) PARP1 binds to the DSB and recruits the MRN complex (MRE11-RAD50-NBS1). (3) The MRN complex exposes microhomologies at the DNA ends. (4) Flap endonuclease 1 (FEN1) removes non-homologous flaps. (5) DNA polymerase theta (Polθ) fills in the gaps using the microhomologies as templates, and DNA ligase III (Lig3) seals the nicks. This error-prone repair mechanism is upregulated in HR-deficient cancers and contributes to genomic instability and therapy resistance. Created using https://biorender.com/ (the figure is licensed under CC BY 4.0).

Microhomology-mediated end joining, also known as alternative end joining (Alt-EJ) or theta-mediated end joining (TMEJ), is an error-prone DNA repair pathway that becomes especially prominent in cancer cells lacking efficient HR. Unlike HR, which is high-fidelity, MMEJ utilizes short microhomologous sequences (typically 2–20 base pairs) flanking DSBs to align DNA ends for repair [116]. This process is inherently mutagenic, often resulting in deletions, insertions, and genomic rearrangements that contribute to genomic instability. The activation of MMEJ serves as a backup DSB repair mechanism that is especially important when pathways like NHEJ or HR are compromised [117]. The key enzyme involved in this pathway is DNA polymerase theta (POLQ), which is crucial for the repair processes and is often upregulated in tumors. POLQ is considered synthetically lethal with several DNA repair genes, including BRCA1 and BRCA2, meaning that loss of both POLQ and BRCA1/2 function leads to cell death [118]. During MMEJ, end resection exposes microhomologous regions, and POLQ binds to the resulting single-stranded DNA overhangs to facilitate repair synthesis. The final ligation involves LIG3-XRCC1 and/or LIG1 [Figure 5]. Because of its error-prone nature, MMEJ leaves characteristic mutational signatures in the genome.

MMEJ contributes to resistance in two ways. First, Polθ-mediated joining at short microhomologies can create the deletions that restore the BRCA reading frame. In TOPARP-B, 60% of the reversions caused by frameshift deletions were flanked by microhomologies, which points to POLQ [102]. A pooled analysis of BRCA reversions found similar breakpoint signatures [110]. Second, PARPi-resistant cells with 53BP1 or Shieldin defects depend on Polθ and are killed by Polθ inhibitors [60].

## 11. Targeting DNA Damage Response to Overcome PARPi Resistance

POLQ inhibitors: POLQ (DNA polymerase theta) has emerged as a promising therapeutic target, especially in the context of PARPi resistance in HR-deficient cancers. POLQ is the central enzyme mediating MMEJ, an error-prone DNA repair pathway that becomes essential for cell survival when HR is compromised, such as in BRCA1/2 mutated tumors. This synthetic lethal relationship means that simultaneous loss of both POLQ and BRCA1/2 function leads to cell death, making POLQ inhibition a strategic approach to selectively kill HR-deficient cancer cells. Preclinical studies have demonstrated that genetic knockout of POLQ impairs survival and proliferation of BRCA1/2-deficient cells [59]. Recent research has focused on developing small-molecule POLQ inhibitors, such as novobiocin, which have been shown to enhance the cytotoxic effects of various PARP inhibitors that induce replication stress [119]. The lethality relationship between POLQ inhibition and HR deficiency remains intact in several models of PARPi resistance, suggesting POLQ inhibitors may be effective in PARPi-resistant tumors. While clinical trials of POLQ inhibitors are still in early phases, preliminary data indicate these agents could address an important resistance mechanism in HR-deficient cancers (Table 2).

Clinical translation of POLQ inhibitors has lagged behind preclinical enthusiasm, and most compounds, including ART558, remain confined to in vitro and xenograft studies with no patients tested yet. Two exceptions stand out. Novobiocin is now in a phase 1 trial testing BRCA-mutant and other DNA repair-deficient solid tumors, open to patients whether or not they have already tried a PARP inhibitor [120]. ART6043 has moved further. It is being tested alongside olaparib in a randomized phase 2 trial (POLKA, NCT05898399) enrolling approximately 80 patients with locally advanced or metastatic BRCA-mutated, HER2-negative breast cancer, randomized to receive either ART6043 plus olaparib or olaparib alone [121]. One possible route for clinical translation is the study of combinations of these agents and PARP inhibitors, especially in patients with progressive disease during PARPi treatment or who do not qualify for it. Patients may be selected based on biomarkers such as BRCA1/2 and other alterations in HR genes. Although the predictive value of these markers for POLQ-targeted combinations requires thorough long-term investigation, given the synthetic lethal relationship between POLQ inhibition and HR deficiency. Toxicity data remain thin, since trials are too early to report mature safety results. Preclinical work shows POLQ inhibition kills HR-deficient cells while sparing normal tissue [59]. This points to a wider therapeutic window than dual PARP1/2 inhibitors typically allow. That selectivity might translate to less myelosuppression in patients, though this is not yet confirmed. POLQ, like PARP1, plays a role in normal-cell DNA repair, so on-target toxicity in fast-dividing tissue such as bone marrow or gut lining is a plausible risk once larger trials report safety data.

The majority of POLQ inhibitors have not failed a clinical trial to date. The real limitation is that almost no clinical data exist yet, not that existing data have disappointed.

Several inhibitors show promise for overcoming PARPi resistance. But their therapeutic potential varies with the resistance mechanism and the level of evidence that is available. POLQ inhibitors such as ART558 target the polymerase activity of Polθ, blocking error-prone end joining. These inhibitors show activity in BRCA-deficient models including those with 53BP1/Shieldin-related PARPi resistance. Novobiocin, which inhibits the ATPase activity of Polθ, also acts in resistant tumor models. However, its development as a targeted therapy requires further evaluation [120]. Another approach is to target translesion synthesis (TLS). JH-RE-06 disrupts the REV1–REV7 interaction and has enhanced olaparib activity in preclinical ovarian cancer models, including organoids and animal models [122,123]. ATR inhibition offers a more clinically advanced strategy. In the phase II CAPRI study, combining ceralasertib with olaparib produced partial responses in 6 of 12 evaluable patients with acquired PARPi-resistant, HR-deficient ovarian cancer. However, the small, single-arm study limits conclusions about efficacy across patient groups [124]. Overall, these findings support targeting compensatory DNA repair and replication-stress pathways, but most still lack definitive evidence of clinical benefit. Biomarker-based patient selection, larger clinical trials and better characterization of treatment-related toxicity will be important for determining which strategies are most likely to benefit patients.

### 11.1. TLS

Another promising strategy to overcome PARPi resistance is targeting the DNA damage tolerance pathway known as translesion synthesis (TLS). TLS is a DNA damage tolerance mechanism that allows cells to bypass replication-blocking lesions using specialized, error-prone DNA polymerases [125]. This process is initiated when the RAD18/RAD6 complex monoubiquitinates proliferating cell nuclear antigen (PCNA) at K164, recruiting TLS polymerases to stalled replication forks. TLS proceeds in two steps: first, Y-family polymerases (Polη, Polι, Polκ, and REV1) insert nucleotides opposite the lesion; second, the B-family Polζ complex (REV3L/REV7/POLD2/POLD3) extends DNA synthesis past the lesion. REV1 acts as a scaffold, coordinating the activity of these polymerases through multiple protein–protein interactions [126,127].

TLS enables cancer cells to survive replication stress and contributes to chemoresistance and mutagenesis. TLS lets replication continue across the lesions and gaps that PARPi leave behind. REV1-Polζ fills PRIMPOL-dependent ssDNA gaps by mutagenic repair, which keeps HR-deficient cells alive [126]. In a patient-derived small cell lung cancer model, upregulated TLS made cells tolerant to olaparib plus temozolomide, and TLS inhibitors restored sensitivity in vitro and in vivo [128]. In BRCA-deficient ovarian cancer models, blocking TLS delayed the appearance of PARPi resistance [123]. TLS polymerases not only resume DNA synthesis at halted forks but also fill in single-stranded DNA gaps left after replication [129,130]. Inhibiting TLS, particularly by targeting REV1 or Polζ, has been shown to sensitize homologous recombination-deficient and PARPi-resistant cancer cells to DNA-damaging agents, making TLS inhibition an attractive therapeutic strategy [125,131,132]. The TLS mechanism is particularly relevant in the context of chemoresistance, as cancer cells can survive and adapt to genotoxic therapies by activating TLS polymerases [132,133].

#### TLS Inhibitors

TLS inhibitors can be broadly classified into two categories: those that directly inhibit the catalytic activity of TLS polymerases and those that disrupt the protein–protein interactions (PPIs) essential for TLS complex assembly [134,135]. While direct catalytic inhibition is challenging due to the conserved structure of polymerase active sites, disrupting PPIs like those involving REV1, REV7, or PCNA offers a more selective approach. Combining TLS inhibitors with standard genotoxic therapies or PARP inhibitors may enhance treatment efficacy and delay resistance by reducing therapy-induced mutagenesis [123,133,136]. Ongoing research and clinical development of TLS inhibitors hold promise for overcoming PARPi resistance and improving outcomes for patients with HR-deficient cancers [Table 3].

Among the TLS inhibitors currently being studied, JH-RE-06 has proven efficacy in both in vivo cancer models and studies of PARPi resistance. JH-RE-06 works by blocking the interaction between REV1 and REV7, thus preventing the recruitment of Polζ and inhibiting mutagenic TLS. The biochemical analysis of JH-RE-06 showed that the IC_50_ value of 0.78 μM was reached for the REV1-REV7 interaction [122]. Previously, it was shown that JH-RE-06 prolonged the sensitivity of cells towards cisplatin and inhibited cancer cell growth in mouse xenograft models. A recent study showed that JH-RE-06 could work in synergy with olaparib in ovarian cancer cells with HR deficiencies as well as in patient-derived organoids. The combination of the two drugs also improved the anti-cancer effect and slowed down or eliminated the appearance of resistance to PARPi in models of xenograft and syngeneic ovarian cancer models [123]. Inhibition of TLS also inhibited the development of mutations in BRCA2, which usually develop as a result of prolonged treatment with olaparib, and proves that TLS helps in the rescue of the replication fork and the emergence of resistance to PARPi. The use of TLS targeting techniques in combination with PARPi has shown positive outcomes, but more research will be needed to understand how safe and effective these new strategies are in the long term [123].

None of the TLS inhibitors in Table 3 have entered clinical trials. All remain in preclinical development, tested only in cell lines, organoids, or xenografts. This gap exists mainly because REV1, REV7, and Polζ interactions are hard to drug with the same precision achieved for catalytic active sites, and because no TLS-specific biomarker yet exists to guide patient selection the way BRCA status does for PARP inhibitors [143].

The more immediate translational route runs through combinations with drugs already in the clinic. TLS activity is mechanistically tied to resistance against ATR and Wee1 inhibitors, both of which are already being tested in patients, as covered above. Cells that stay TLS-proficient resist ATR and Wee1 inhibition, and adding a REV1-targeting TLS inhibitor restores sensitivity to both in preclinical models [136]. This suggests TLS inhibitors are more likely to reach patients first as an add-on to an existing ATR or Wee1 inhibitor regimen than as a standalone drug.

### 11.2. Epigenetic Modifications

Epigenetic alterations can significantly impact DNA repair capacity and PARPi response. BRCA1 promoter hypermethylation, which silences BRCA1 expression, can create a BRCAness phenotype and sensitivity to PARPi. Histone modifications, particularly those affecting chromatin accessibility, also influence DNA repair pathway choice and PARPi response. PARPi treatment itself can induce changes in the chromatin landscape, notably elevated H3K9me2, a hallmark of facultative heterochromatin [114]. This PARPi-induced heterochromatin formation increases dependence on ATR activity for replication fork progression, providing a rationale for ATRi and PARPi combination therapy [144]. Understanding these epigenetic mechanisms has the potential to lead to better patient selection and a greater understanding of the causes of treatment resistance, which, in turn, could facilitate the development of approaches to circumvent or prevent epigenetically mediated resistance.

### 11.3. ATR Inhibitors

ATR (Ataxia telangiectasia and Rad3-related) is a master kinase in the DNA damage response, particularly responsive to replication stress, ssDNA regions, stalled replication forks, resected DSBs and other DNA lesions that arise during DNA replication and repair. Upon DNA damage, ATR is recruited to sites of extensive ssDNA coated with replication protein A (RPA), where it forms a complex with its partner ATRIP. Activation of ATR is mediated by proteins such as TOPBP1 and ETAA1, which facilitate ATR’s kinase activity through distinct mechanisms [145,146,147,148]. Once activated, ATR phosphorylates downstream effectors like Chk1, leading to cell cycle arrest and allowing time for DNA repair.

ATR inhibitors have become popular in recent years and are currently in preclinical and clinical development. These inhibitors can be used as monotherapy or in combination with other treatments to abolish replication stress-induced cell cycle checkpoints and promote premature mitotic entry of damaged cells. Mechanistically, ATR inhibition prevents Chk1 activation, disrupts the DNA damage-induced checkpoint, and forces cells with damaged DNA into mitosis, resulting in cell death [149]. By targeting the ATR pathway, these inhibitors sensitize cancer cells to DNA-damaging agents and can overcome resistance mechanisms similar to those seen with PARP inhibitors.

Tumor selection for ATR inhibitor trials is increasingly guided by DNA damage response biomarkers rather than histology alone. ATM loss or mutation is the most extensively studied biomarker: the phase IIa PLANETTE study tested ceralasertib monotherapy in two ATM-altered cohorts, 30 patients with advanced solid tumors and 15 with metastatic castration-resistant prostate cancer, all with centrally confirmed ATM alterations and prior standard treatment [150]. In the phase 2 HUDSON umbrella trial in non-small-cell lung cancer, durvalumab plus ceralasertib outperformed three other DDR-targeted combinations, and the ATM-altered subgroup achieved a 26.1% objective response rate with a median overall survival of 22.8 months, compared with 13.9% and 17.4 months in the unselected durvalumab–ceralasertib cohort [151]. Eligibility is expanding beyond ATM: the phase 1 TRESR trial enrolled 120 patients with advanced solid tumors selected for loss-of-function alterations across a broader DDR gene panel, including BRCA1/2, CDK12, MRE11, PALB2, RAD51 paralogs, and RNASEH2A/B [152]. Additional trial evidence spans hepatocellular carcinoma [153], small cell lung cancer with high replication stress [154], and triple-negative breast cancer in combination with cisplatin [155], with ATM-deficient prostate cancer proposed as a rational setting for combined PARP and ATR inhibition [156]. Nearly all current ATR inhibitor trials enroll patients with advanced, metastatic, previously treated disease rather than treatment-naive primary tumors, positioning ATR inhibitors as later-line, biomarker-selected therapy rather than first-line treatment. Table 4 below indicates various ATR inhibitors which are currently in preclinical and clinical development.

### 11.4. In Vivo Assays and Efficacy

Various in vivo models have exhibited the effectiveness of ATR inhibitors. Among them are syngeneic mice (Hepa1-6 liver tumors in mice) and human tumor xenografts. Notably, AZD6738 plus radiation in short courses resulted in an increase in selective CD8^+^ effector T cells, resulting in tumor control [153,157]. Additionally, in xenograft models, combinations of ATR inhibitors also inhibited tumor growth, as in H82/H446 SCLC xenograft models in which treatment with oral M4344 and irinotecan resulted in almost complete tumor regression and a significant increase in survival compared to the results shown by other agents [158] (Table 4). Results show that ATR inhibitors convert resistant tumors into sensitive ones by preventing the DNA repair and checkpoint response.

## 12. Current Clinical Biomarkers for PARPi Response

Recent studies such as SOLO-1 (NCT01844986) and OlympiAD (NCT02000622) have proven that BRCA1/2 mutations are a good predictor of the effectiveness of PARPi. It was confirmed that mutations in HR repair genes like PALB2, RAD51C, or RAD51D lead to sensitivity to PARPi [119,161,162]. The POLO and PROfound trials showed a positive response of PARPi in patients with PALB2 deletion in pancreatic cancer. Multi-gene panels using next-generation sequencing (NGS) are increasingly being used for identifying alterations in DDR. Comprehensive molecular profiling should differentiate germline and somatic mutations and take into account their pathogenic significance, the clinical evidence available for the respective gene, and the functional role of the mutation. Even if a mutation is observed within the DDR genes, this does not directly indicate responsiveness to PARPi, as a personalized treatment plan should be based on the relevant scientific evidence, regulatory approvals, and clinical guidelines [163,164].

Genomic HRD score is also seen to be one of the emerging biomarkers that indicate earlier genomic scarring resulting from HRD. Commercial tests like Myriad myChoice^®^ determine the loss of heterozygosity (LOH), telomeric allelic imbalance (TAI), and large-scale transitions (LST) and determine the HRD score using these variables [165,166]. They also correlate with PARPi susceptibility, but with several drawbacks. For instance, many tumors evaluated as HRD-positive still exhibit some HR activity. For instance, tumors assessed to be HRD-positive may induce compensatory RAD51 recruitment and re-establish activity through demethylation of silenced BRCA1. However, HRD genomic scars are far from predictive, as negative responses to treatment can occur in some of the HRD-positive patients [67,167].

Another type of biomarker includes the functional or expression markers. These are used to test BRCA1 promoter methylation by using methylation-specific PCR or NGS, as promoter methylation silences the BRCA1 gene. Resumption of the methylation may result in restoration of HR and lead to resistance [168,169]. Similarly, SLFN11 (Schlafen-11) has emerged as a biomarker. High levels of SLFN11 are associated with replication stress-induced cell death; on the other hand, its absence allows the fork to progress with the help of PARP and confers resistance. As a result, various tests for mutations, such as the HRD score, and functional markers such as SLFN11 or RAD51 foci formation can be used to predict those patients who are likely to be responsive to PARPi [170,171].

### 12.1. Emerging Inhibitors to Overcome PARPi Resistance

One important strategy is to block the cell-cycle checkpoint kinases that HR-proficient cells use to withstand PARPi-induced damage. For instance, ATR inhibitors such as ceralasertib (AZD6738) and berzosertib (VX-970) inhibit the ATR-mediated replication stress response, which prevents repair of lesions caused by PARPi [144]. Likewise, WEE1 inhibitors such as adavosertib (AZD1775) eliminate the G_2_/M checkpoint and force cells with unrepaired DNA to proceed with fatal mitosis [172]. Moreover, CHK1 inhibitors like prexasertib also inhibit the checkpoints during S phase. Several preclinical studies reveal that inhibiting ATR, CHK1, or WEE1 results in creating an HRD state that leads to synthetic lethality in combination with PARPi [173].

Inhibitors of POLθ target MMEJ, which is often used by HRD tumors. Since POLQ is synthetically lethal with BRCA deficiency, POLQ inhibitors such as novobiocin derivatives can kill PARPi-resistant cells. By blocking POLQ, the cells become sensitive again to PARPi treatment. Similarly, cell-cycle/CDK inhibitors such as CDK1/2/9 inhibitors can downregulate HR repair factors [60,174]. It has been found in preclinical studies that CDK7/9 inhibitors prevent RAD51 from loading, while CDK4/6 inhibitors such as palbociclib can be used together with PARPi in resistant cancer models [175,176]. Although there are several emerging inhibitors targeting PARPi resistance, continued clinical trials and biomarker-based studies are required to refine which patients specifically benefit from each of the combinations.

### 12.2. Limitations

While PARP inhibitors have transformed the treatment of HR-deficient cancers, several limitations remain. Predictive biomarkers such as BRCA mutation status and HRD scores are not fully reliable, as not all patients respond and resistance frequently develops. The mechanisms of acquired resistance including HR restoration, drug efflux, replication fork protection, and activation of alternative repair pathways limit the long-term efficacy of PARPi. Furthermore, many novel inhibitors and combination strategies are still in early clinical development, with their safety and effectiveness yet to be established. The integration of advanced molecular diagnostics into routine care is also challenged by issues of accessibility and standardization. Addressing these limitations is essential for improving patient selection, overcoming resistance, and optimizing outcomes in PARPi-based cancer therapy.

## 13. Future Directions

Advances in molecular diagnostics are enabling more precise selection of patients for PARP inhibitors and DDR-targeted therapies. Genomic profiling for HRD scores, BRCA1/2 status, and other DDR gene alterations helps identify those most likely to benefit from PARPi. Emerging biomarkers, such as RAD51 foci and circulating tumor DNA, may further refine patient selection [109,177] and guide adaptive treatment strategies, maximizing efficacy and minimizing unnecessary toxicity. Next-generation PARP inhibitors are being developed with enhanced PARP trapping ability, improved selectivity, and reduced off-target effects. New agents targeting other DDR components such as POLQ, ATR, and DNA-PK are in preclinical and early clinical trials [178]. These novel inhibitors offer hope for overcoming acquired resistance and expanding the benefits of DDR-targeted therapy to a broader patient population. Combining PARP inhibitors with immune checkpoint inhibitors is an emerging strategy to enhance antitumor immunity. PARPi can increase tumor immunogenicity by activating the cGAS-STING pathway and promoting interferon signaling, thereby sensitizing tumors to immunotherapy [179]. Early clinical trials of PARPi and immune checkpoint blockade combinations show positive outcomes, particularly in HR-deficient and immunologically “cold” tumors, and ongoing studies aim to optimize these regimens for durable clinical benefit [180]. As PARP inhibitors move into earlier lines of therapy and broader patient populations, understanding the molecular determinants of resistance is critical for optimizing outcomes. Incorporating biomarker-based approaches with combination strategies may offer opportunities to better understand treatment resistance and identify potential strategies for improving patient outcomes.

## 14. Conclusions

PARP inhibitors have revolutionized the treatment of homologous recombination-deficient cancers, particularly those with BRCA1/2 mutations, by exploiting synthetic lethality and targeting the DNA repair machinery. However, resistance to these agents remains a significant clinical challenge, arising through mechanisms such as restoration of homologous recombination, replication fork protection, and activation of alternative repair pathways like microhomology-mediated end joining and translesion synthesis. As depicted in the accompanying figures, PARP plays a central role in base excision repair, and its inhibition leads to failed repair, fork collapse, and cell death in HR-deficient cells, while also highlighting the compensatory activation of error-prone pathways like MMEJ. Ongoing progress in the use of inhibitors directed against POLQ, TLS polymerases, and ATR, as well as the exploration of possible combinations of therapeutic agents and molecular diagnostic methods, may provide insights on their possible functions in fighting drug resistance and on how to utilize them in making treatment decisions.

Continued research into the dynamic interplay of DNA repair pathways and the identification of robust predictive biomarkers will be essential for optimizing the long-term efficacy of PARP inhibitors and extending their benefits to a broader spectrum of cancer patients.

## Figures and Tables

**Table 2:** Summary of selected DNA repair inhibitors.

Name of the Inhibitor	Mechanism of Action and Mode of Study	Route of Administration	Inhibitory Concentration (IC50)	Clinical Status	Reference
Novobiocin	Synthetically lethal in HR-deficient tumors and PARPi resistance models	Oral (preclinical)	~15–50 μM (Biochemical assay)	Phase 1 trial in BRCA-mutant and other DNA damage repair-deficient solid tumors, open to both PARP inhibitor-naive and PARP inhibitor-resistant patients	[59,120]
ART558	Selective POLQ inhibitor; synthetic lethal with HR deficiency along with PARPi in both breast and ovarian cancer cells and in 3D organoid models	Not specified (preclinical)	~7.9 nM (Biochemical assay)	Preclinical only; no registered clinical trials to date	[60]
Camptothecin	Topoisomerase I inhibitor; induces DNA SSBs, used to study synergy with POLQ knockout and PARPi resistance; in vitro and in vivo studies	Intravenous (IV), oral (clinical/preclinical)	~0.01–1 μM (Cellular cytotoxicity assay)	Long approved clinically in derivative forms (irinotecan, topotecan); not tested specifically as a POLQ-combination agent	[60]
Etoposide	Topoisomerase II inhibitor; frequently used in combination with POLQ or PARP inhibitors in cell viability assays; in vitro and in vivo studies	Intravenous (IV), oral	~0.1–10 μM (Cellular cytotoxicity assay)	Long approved clinically; used investigationally alongside POLQ/PARP inhibitors in preclinical studies, but no dedicated POLQ-combination trial registered	[60]

Inhibitors are listed with mechanism of action and mode of study, route of administration, reported IC50 (half-maximal inhibitory concentration, as given in the cited studies), and clinical status. ART6043 is being tested in the phase 2 POLKA trial (NCT05898399) [121]; its preclinical potency has not been disclosed and is not listed. HR, homologous recombination; PARPi, PARP inhibitor.

**Table 3:** Summary of key TLS inhibitors. Inhibitors are listed with TLS target, structure, function, reported IC50 or Kd, model system (in vitro and/or in vivo) and cancer type. TLS, translesion synthesis; PPI, protein–protein interaction; Kd, dissociation constant; MST, microscale thermophoresis; SPR, surface plasmon resonance.

TLS Inhibitor	Molecular Target	Chemical Structure	Mechanism of Action	IC_50_/K_d_	Evidence	Investigated Types of Cancer	Reference
Phenazopyridine (PAP) analog 1	REV1 (RIR interface)	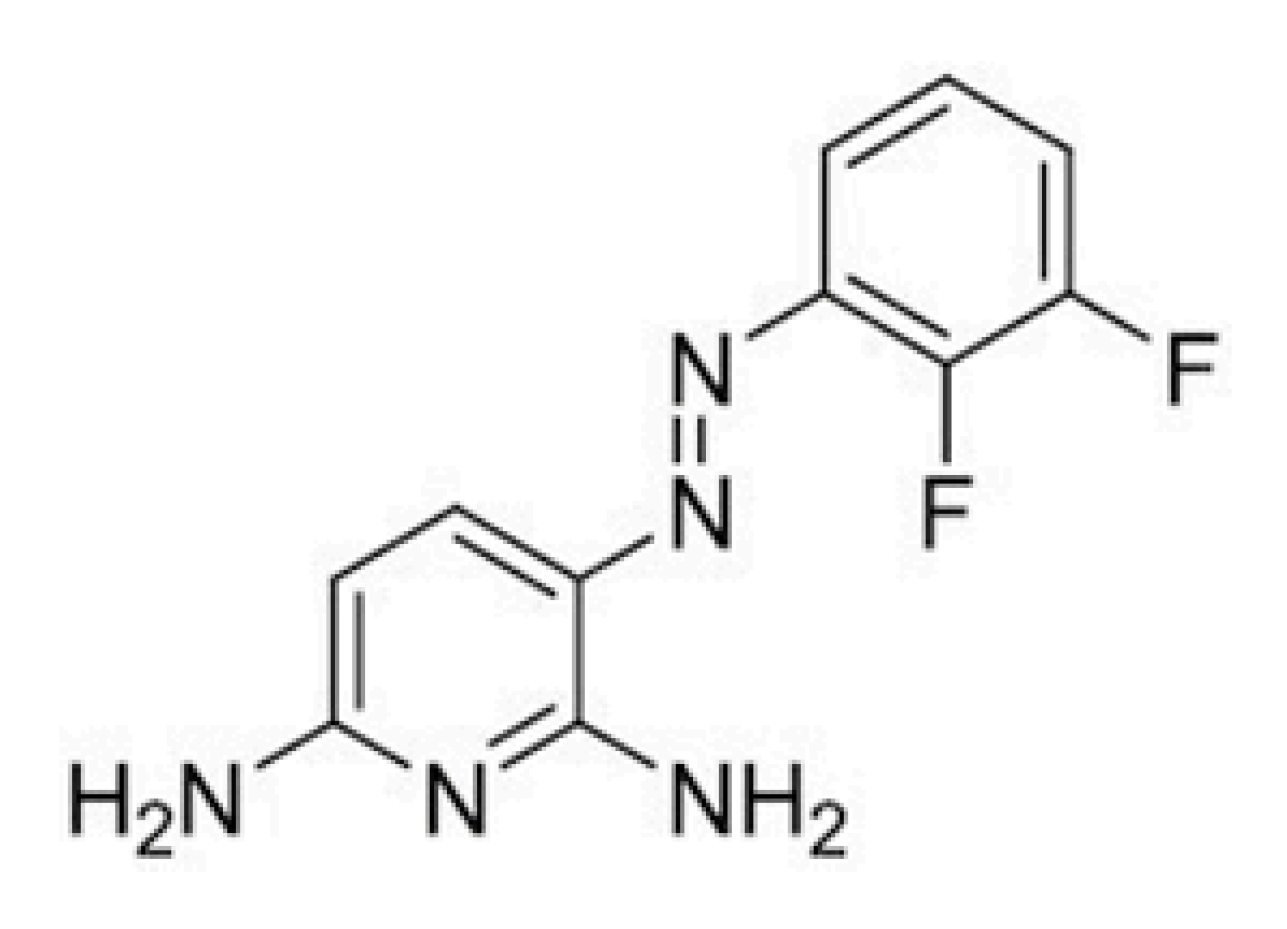	Disrupts interactions between REV1-CT and the REV1-interacting regions (RIRs) of TLS polymerases	*K*_d_ = 21 μM (MST)	Both	Small cell lung cancer	[128]
JH-RE-06	Rev1-Rev7 interface	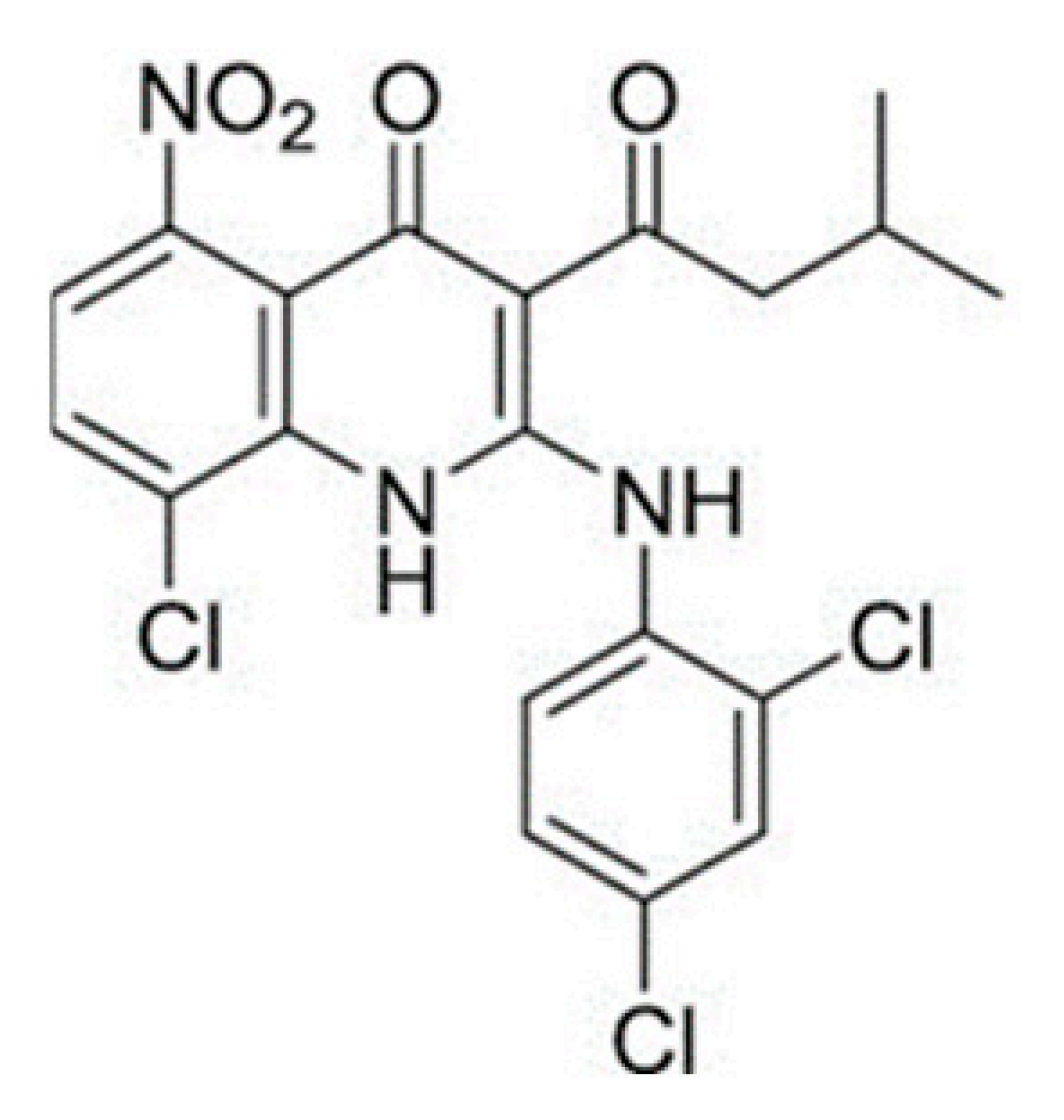	Disrupts the REV1-REV7 interaction, interfering with recruitment of Pol ζ during TLS	0.78 µM	Both	Breast, ovarian cancer	[122,137]
MLAF50	REV1 UBM2 (ubiquitin interface)	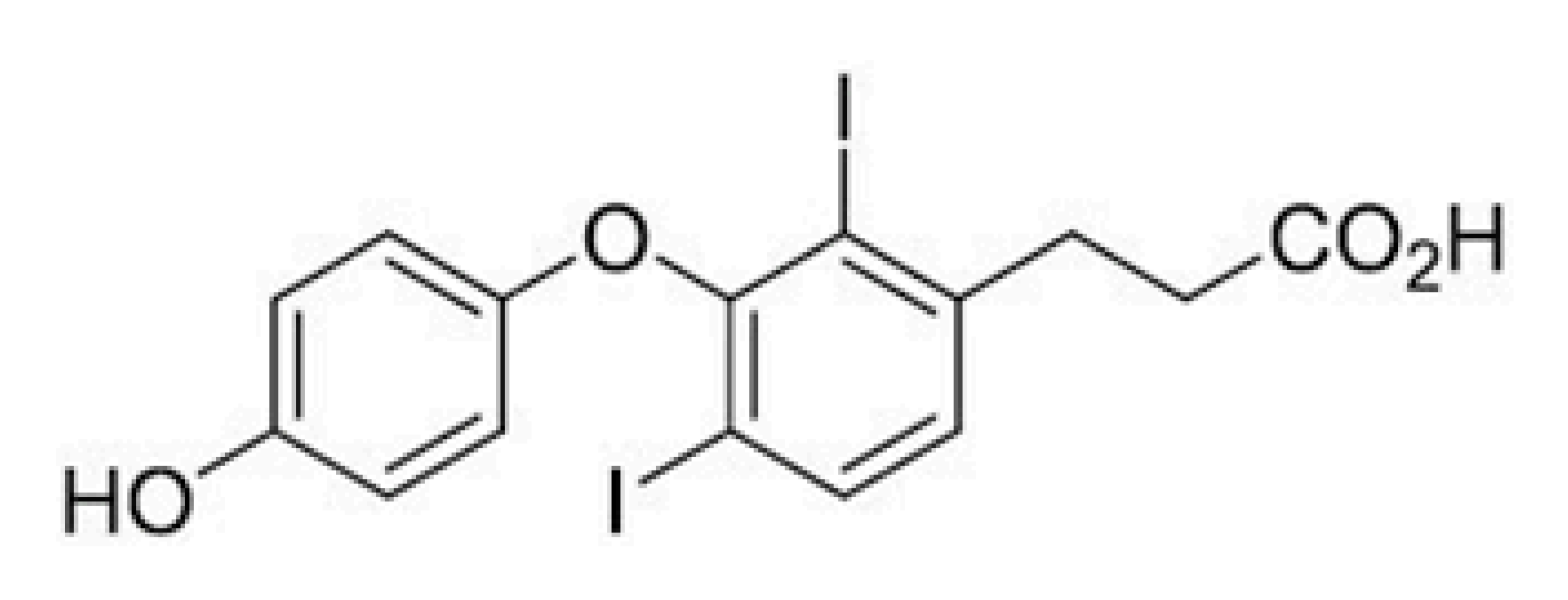	Binds REV1 UBM2 and interferes with the REV1–ubiquitin interaction	*K*_d_ = 37 μM (SPR)	Only in vitro	Human osteosarcoma (U2OS cell model)	[138]
Compound 7	REV7	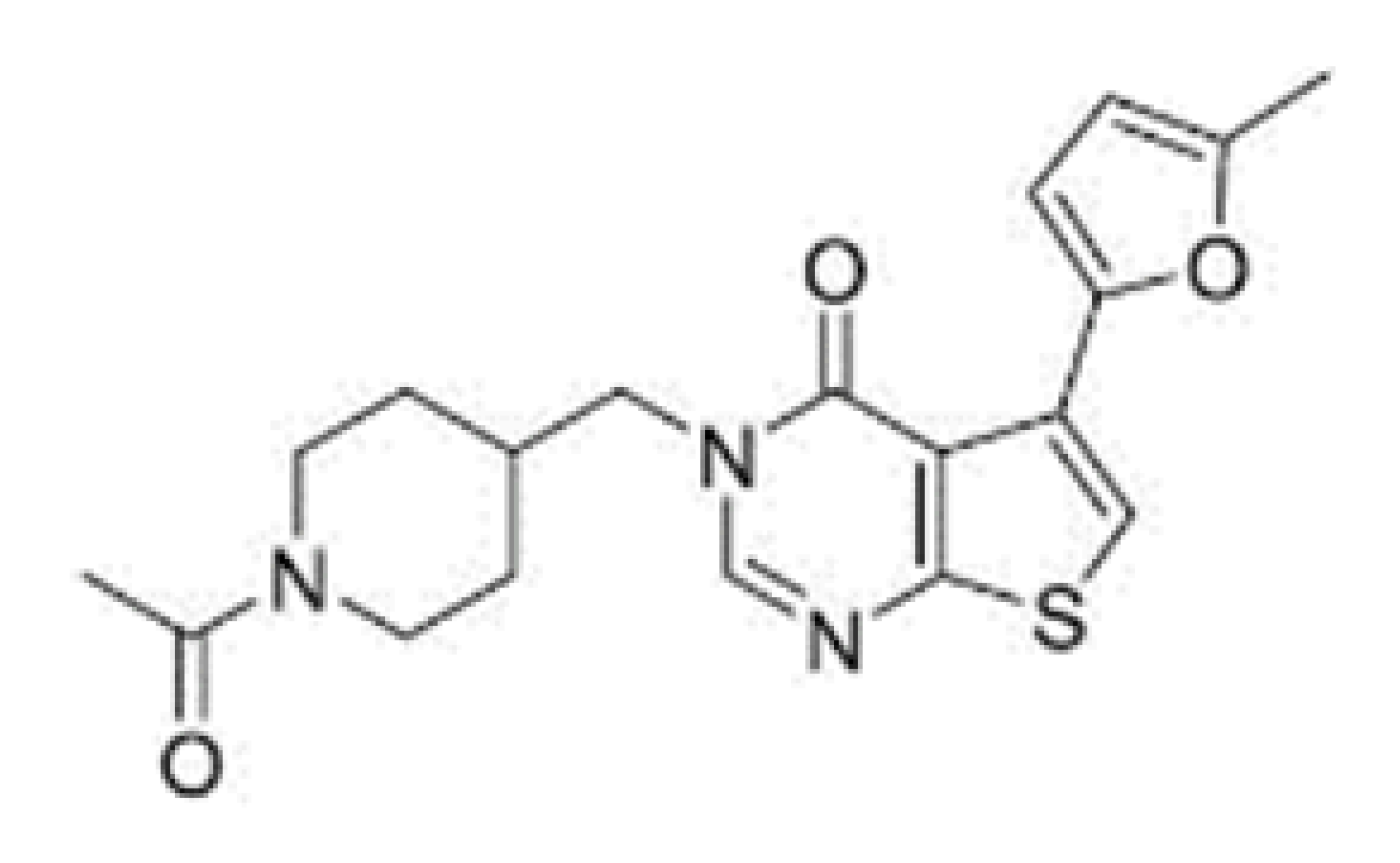	Binds to REV7 and disrupts its interaction with REV3L, inhibiting Pol ζ mediated DNA interstrand crosslink repair	IC_50_ = 78 μM	Only in vitro	HeLa cells (cervical cancer)	[139]
PNR-7-02	Pol eta/REV1	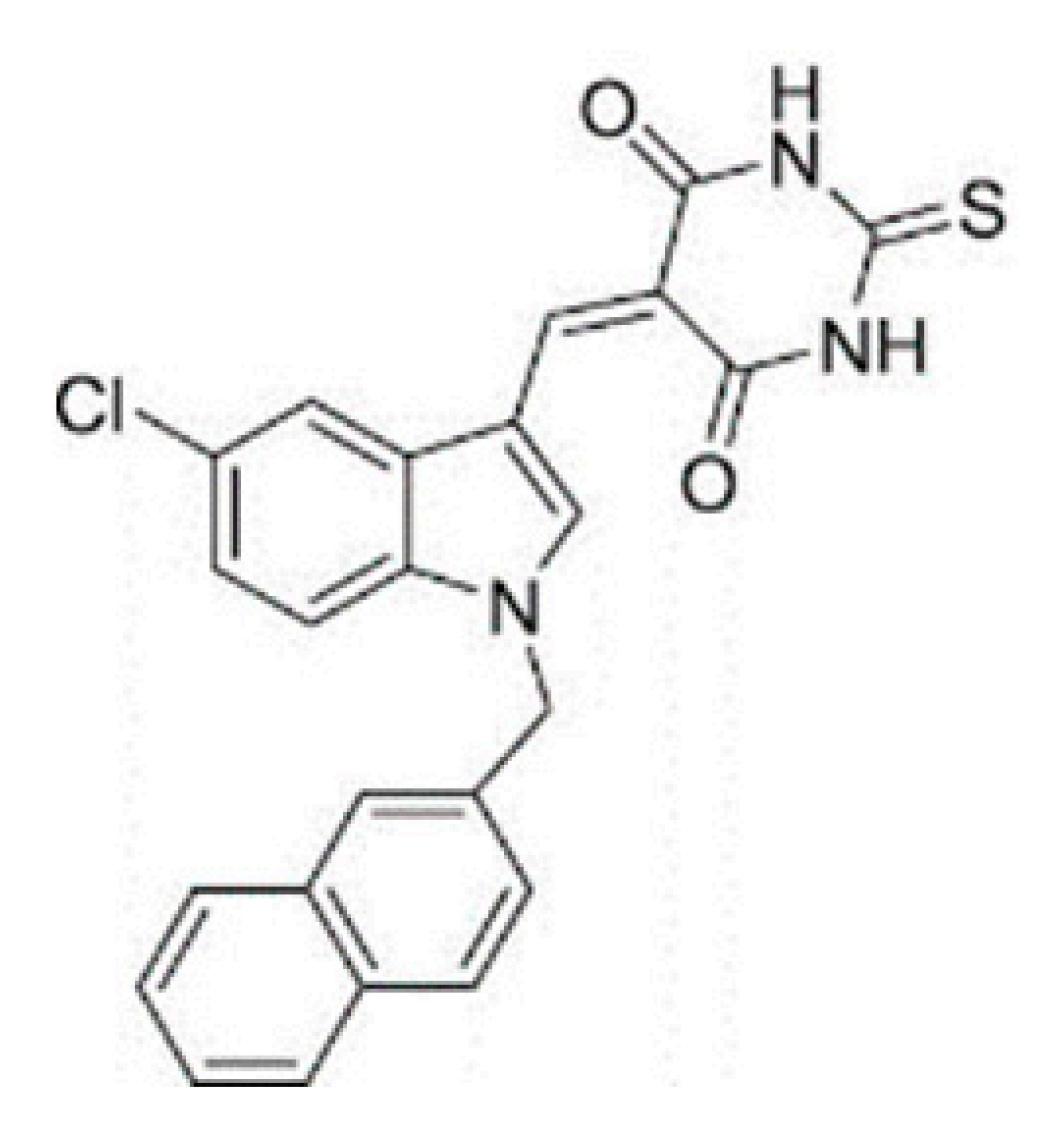	Inhibits the polymerase activity of Pol η, REV1, and Pol λ at approximately 8 µM; shows lower potency against Pol κ	IC_50_ = 8.0 μM	Only in vitro	Chronic myelogenous leukemia-derived HAP1 cells	[140]
IAG-10	Pol kappa	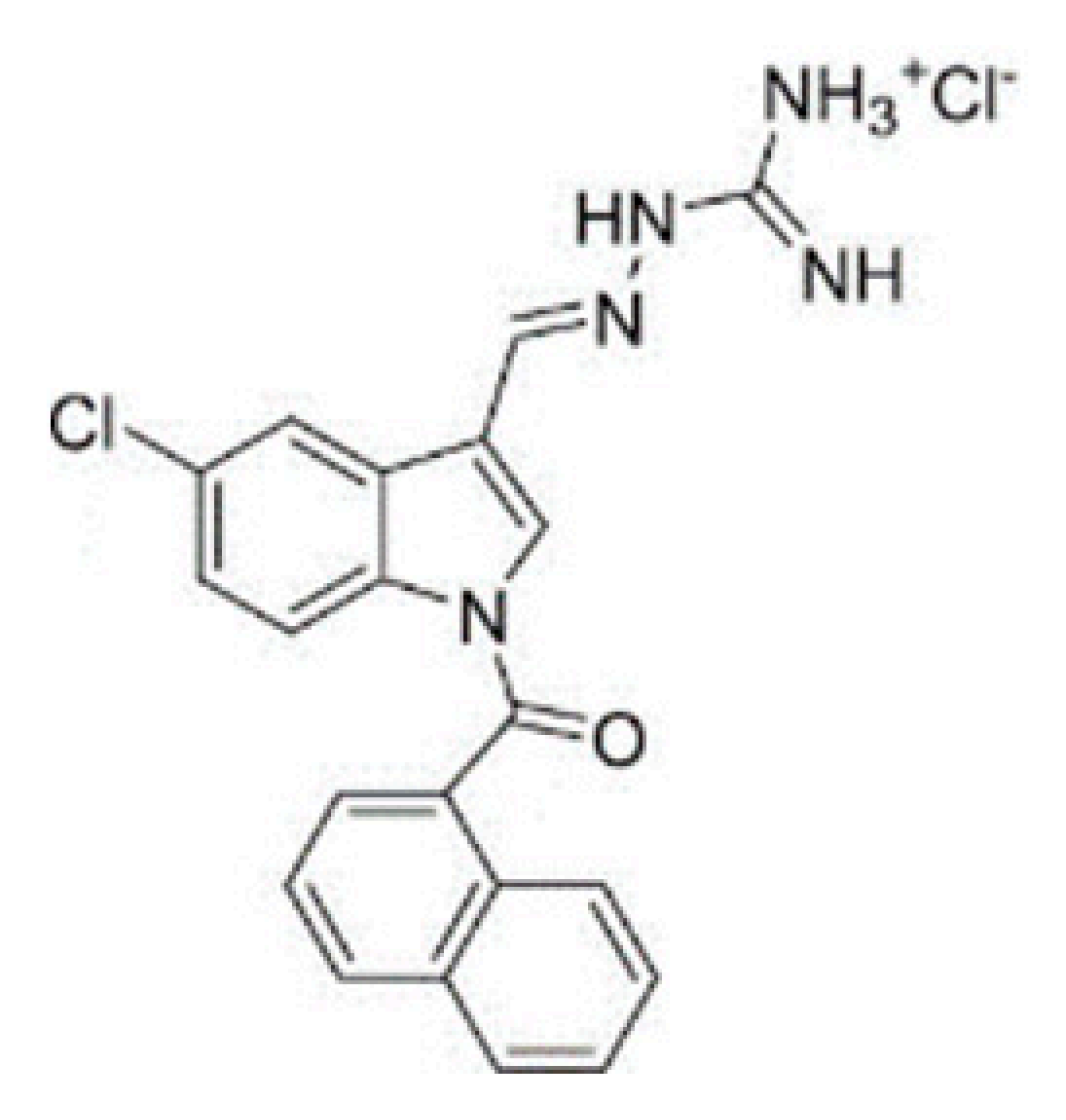	Selectively inhibits human Pol κ activity	IC_50_ = 0.7–7 μM	Only in vitro	HAP-1 cells	[141]
T2AA	PCNA	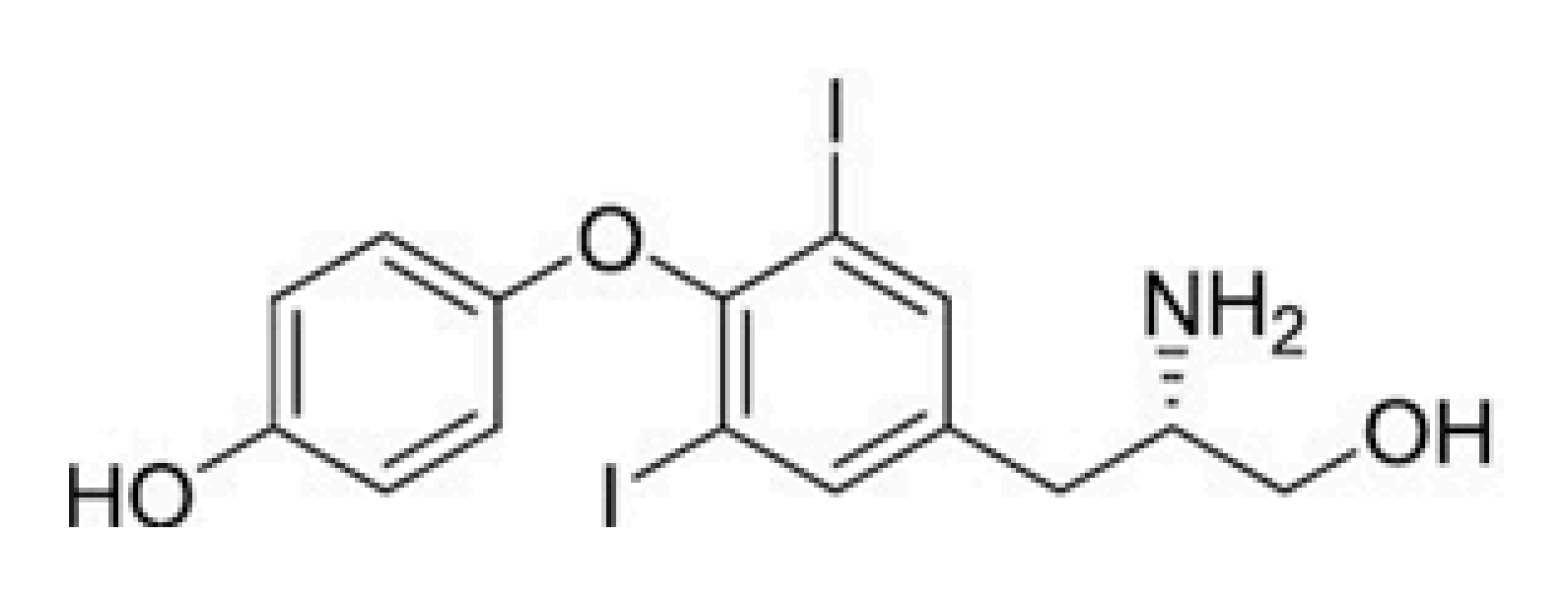	Disrupts PCNA-mediated interactions with Pol η and REV1	IC_50_ = 1 μM	Only in vitro	HeLa and U2OS cells	[142]

**Table 4:** Overview of key ATR inhibitors in clinical and preclinical studies.

ATR Inhibitor (Company)	Mechanism of Action	Experimental Models	Cancer Type(s)	Reference
AZD6738 (Ceralasertib)	ATR kinase inhibitor; induces replication stress and sensitizes tumors to radiotherapy. Shows synergistic antitumor activity with radiotherapy and immunotherapy in preclinical models.	In vivo syngeneic mouse models (Hepa1-6 murine HCC in C57BL/6 and BALB/c); liver cancer model.	Hepatocellular carcinoma (mouse HCC)	[153,157]
BAY 1895344 (Elimusertib)	ATR inhibitor; mild toxicity as single agent. Synergizes with PARP inhibitors and ATM kinase blockade. Enhances DNA damage and cell death in ATM-deficient or repair-compromised contexts.	In vitro prostate cancer cell lines (22RV1, LNCaP, DU-145), ATM-knockout cells and 3D organoids; in vivo prostate xenografts.	Prostate cancer (and other DDR-deficient cancers)	[156]
M4344 (VX-803)	Potent ATR inhibitor; causes DNA damage. Strong synergy with topoisomerase I inhibitors (topotecan/irinotecan). Enhances cytotoxicity of many chemotherapies.	In vitro cancer cell lines (various histologies) and patient-derived organoids; in vivo xenografts (e.g., H82/H446 SCLC models).	Small-cell lung cancer (and other replication-stressed tumors)	[158]
Berzosertib (M6620)	ATR inhibitor; blocks ATR-CHK1 checkpoint, preventing repair of therapy-induced DNA damage. Synergizes with topoisomerase I inhibitors (topotecan) in vitro and in vivo. Enhances efficacy in platinum-resistant settings.	In vitro SCLC cell lines (DMS114, H446); in vivo Colo205 colorectal xenografts; Phase 2 trial in relapsed SCLC.	Platinum-resistant small-cell lung cancer	[154,155]
RP-3500 (Camonsertib)	Selective ATR inhibitor; induces replication stress and DNA damage as monotherapy. Shows broad single-agent activity in DDR-deficient models and tumor xenografts.	In vitro DDR-deficient cancer cell lines; in vivo patient-derived and cell line xenografts (colon, ovarian, breast, pancreatic, prostate).	Diverse solid tumors (colon, ovarian, breast, pancreatic, prostate)	[159]
ART0380	Potent oral ATR inhibitor; monotherapy cytotoxicity and combination with DNA-damaging agents. Being tested in various tumors (trial NCT04657068).	In vitro cancer cell lines and xenografts; ongoing Phase 2 clinical trials in multiple tumor types.	Multiple tumor types (solid tumors)	[160]

## Data Availability

No new data were created or analyzed in this study. Data sharing is not applicable to this article.
